# Aberrant elevation of FTO levels promotes liver steatosis by decreasing the m^6^A methylation and increasing the stability of SREBF1 and ChREBP mRNAs

**DOI:** 10.1093/jmcb/mjac061

**Published:** 2022-11-10

**Authors:** Zhili Tang, Chao Sun, Ying Yan, Zhoumin Niu, Yuying Li, Xi Xu, Jing Zhang, Yuting Wu, Yan Li, Li Wang, Cheng Hu, Zhuoyang Li, Jingjing Jiang, Hao Ying

**Affiliations:** CAS Key Laboratory of Nutrition, Metabolism and Food Safety, Shanghai Institute of Nutrition and Health, University of Chinese Academy of Sciences, Chinese Academy of Sciences, and Shanghai Jiao Tong University Affiliated Sixth People's Hospital, Shanghai 200031, China; Innovation Center for Intervention of Chronic Disease and Promotion of Health, Shanghai 200031, China; CAS Key Laboratory of Nutrition, Metabolism and Food Safety, Shanghai Institute of Nutrition and Health, University of Chinese Academy of Sciences, Chinese Academy of Sciences, and Shanghai Jiao Tong University Affiliated Sixth People's Hospital, Shanghai 200031, China; CAS Key Laboratory of Nutrition, Metabolism and Food Safety, Shanghai Institute of Nutrition and Health, University of Chinese Academy of Sciences, Chinese Academy of Sciences, and Shanghai Jiao Tong University Affiliated Sixth People's Hospital, Shanghai 200031, China; CAS Key Laboratory of Nutrition, Metabolism and Food Safety, Shanghai Institute of Nutrition and Health, University of Chinese Academy of Sciences, Chinese Academy of Sciences, and Shanghai Jiao Tong University Affiliated Sixth People's Hospital, Shanghai 200031, China; CAS Key Laboratory of Nutrition, Metabolism and Food Safety, Shanghai Institute of Nutrition and Health, University of Chinese Academy of Sciences, Chinese Academy of Sciences, and Shanghai Jiao Tong University Affiliated Sixth People's Hospital, Shanghai 200031, China; Department of Endocrinology and Metabolism, Zhongshan Hospital, Fudan University, Shanghai 200031, China; Department of Endocrinology and Metabolism, Zhongshan Hospital, Fudan University, Shanghai 200031, China; CAS Key Laboratory of Nutrition, Metabolism and Food Safety, Shanghai Institute of Nutrition and Health, University of Chinese Academy of Sciences, Chinese Academy of Sciences, and Shanghai Jiao Tong University Affiliated Sixth People's Hospital, Shanghai 200031, China; State Key Laboratory of Food Science and Technology, School of Food Science and Technology, Jiangnan University, Wuxi 214122, China; State Key Laboratory of Food Science and Technology, School of Food Science and Technology, Jiangnan University, Wuxi 214122, China; Shanghai Diabetes Institute, Shanghai Key Laboratory of Diabetes Mellitus, Shanghai Clinical Centre for Diabetes, Shanghai Jiao Tong University Affiliated Sixth People's Hospital, Shanghai 200233, China; CAS Key Laboratory of Nutrition, Metabolism and Food Safety, Shanghai Institute of Nutrition and Health, University of Chinese Academy of Sciences, Chinese Academy of Sciences, and Shanghai Jiao Tong University Affiliated Sixth People's Hospital, Shanghai 200031, China; Department of Endocrinology and Metabolism, Zhongshan Hospital, Fudan University, Shanghai 200031, China; CAS Key Laboratory of Nutrition, Metabolism and Food Safety, Shanghai Institute of Nutrition and Health, University of Chinese Academy of Sciences, Chinese Academy of Sciences, and Shanghai Jiao Tong University Affiliated Sixth People's Hospital, Shanghai 200031, China; Innovation Center for Intervention of Chronic Disease and Promotion of Health, Shanghai 200031, China; Key Laboratory of Food Safety Risk Assessment, Ministry of Health, Beijing 100021, China

**Keywords:** FTO, m^6^A, SREBF1, ChREBP, lipogenesis, NAFLD

## Abstract

Previous studies have indicated an association of fat mass and obesity-associated (FTO) with nonalcoholic fatty liver disease (NAFLD), the most common chronic liver disease worldwide. This study aimed to decipher the complex role of FTO in hepatic lipid metabolism. We found that a decrease in N^6^-methyladenosine (m^6^A) RNA methylation in the liver of mice fed with a high-fat diet (HFD) was accompanied by an increase in FTO expression. Overexpression of FTO in the liver promoted triglyceride accumulation by upregulating the expression of lipogenic genes. Mechanistical studies revealed that FTO could stabilize the mRNAs of sterol regulatory element binding transcription factor 1 (SREBF1) and carbohydrate responsive element binding protein (ChREBP), two master lipogenic transcription factors, by demethylating m^6^A sites. Knockdown of either SREBF1 or ChREBP attenuated the lipogenic effect of FTO, suggesting that they are *bona fide* effectors for FTO in regulating lipogenesis. Insulin could stimulate FTO transcription through a mechanism involving the action of intranuclear insulin receptor beta, while knockdown of FTO abrogated the lipogenic effect of insulin. Inhibition of FTO by entacapone decreased the expression of SREBF1, ChREBP, and downstream lipogenic genes, ameliorating liver steatosis in HFD-fed mice. Thus, our study established a critical role of FTO in both the insulin-regulated hepatic lipogenesis and the pathogenesis of NAFLD and provided a potential strategy for treating NAFLD.

## Introduction

Nonalcoholic fatty liver disease (NAFLD) has emerged as the most common chronic liver disease, with a global average prevalence ∼25% in adults, higher than type 2 diabetes and obesity (Powell et al., 2021). It is also the fastest-growing cause of liver-related mortality worldwide, currently with no FDA-approved medication (Paik et al., 2020). The pathogenesis of NAFLD has been extensively studied; however, the underlying mechanism remains inconclusive. The hallmark of NAFLD is an increase in hepatic triglyceride (TG) content. A series of studies suggest that increased TG production rather than decreased mobilization is the primary mechanism for the development of NAFLD (Sanyal et al., 2001; Adiels et al., 2005; Bugianesi et al., 2005; Fabbrini et al., 2008; da Silveira et al., 2020). Many lipogenic enzymes, such as adenosine triphosphate citrate lyase (ACLY), acetyl-CoA carboxylase 1 (ACC1), fatty acid synthase (FASN), and stearoyl-coenzyme A desaturase 1 (SCD1), are coordinately regulated by key transcription factors, including sterol regulatory element binding protein 1c (SREBP1c, encoded by *SREBF1*) and carbohydrate responsive element binding protein (ChREBP) (Wang et al., 2015c). It is noteworthy that the insulin effect on both SREBF1 and ChREBP expression has been noticed before, although the underlying mechanism remains unclear.

N^6^-methyladenosine (m^6^A) is the most abundant epigenetic modification found in at least a quarter of mammalian mRNAs, fine-tuning mRNA metabolism in various physiological and pathophysiological processes (Desrosiers et al., 1974; Hsu et al., 2017; Roundtree et al., 2017). In mammals, m^6^A modification is dynamically controlled by a group of enzymes named ‘writers’ (methyltransferase) and ‘erasers’ (demethylase). In general, the methylation is catalyzed by complexes including methyltransferase-like 3 (METTL3), METTL14, and Wilms' tumor 1-associating protein (Liu et al., 2014a), while the demethylation is processed by fat mass and obesity-associated (FTO) and ALKBH5 (Jia et al., 2011; Zheng et al., 2013). YTH domain-containing family protein 1/2/3 (YTHDF1/2/3) (Wang et al., 2015b; Du et al., 2016; Shi et al., 2017), referred to as ‘readers’, can recognize m^6^A. Recent evidence suggests that m^6^A methylation may play a critical role in the regulation of hepatic TG metabolism (Kang et al., 2018; Zhou et al., 2021).

FTO has long been associated with obesity according to early genome-wide association studies (Scuteri et al., 2007) and plays a regulatory role in adipogenesis (Zhao et al., 2014; Wang et al., 2015a). Mice with the whole-body knockout or loss-of-function mutation of FTO had reduced adipose tissue and body weight (BW), while transgenic mice overexpressing FTO displayed increased food intake and obesity (Fischer et al., 2009; Church et al., 2010; McMurray et al., 2013). FTO expression increased in the fatty liver of rats and patients with nonalcoholic steatohepatitis (Guo et al., 2013; Lim et al., 2016). It has been suggested that FTO regulates lipogenesis through modulating SREBP1c activity via multiple mechanisms, affecting nuclear translocation, maturation, as well as m^6^A methylation (Chen et al., 2018; Hu et al., 2020). Nevertheless, the physiological role of hepatic FTO during the fasting–feeding cycle and the pathophysiological role of FTO in diet-induced hepatic steatosis in mice remains unclear.

Here, we demonstrated that hepatic FTO could demethylate and stabilize SREBF1 and ChREBP mRNAs, increasing both SREBP1c and ChREBP protein levels and consequently upregulating the expression of key lipogenic enzymes responsible for hepatic lipogenesis. Moreover, insulin could induce the expression of hepatic FTO, which in turn mediated the lipogenic effect of insulin. Furthermore, inhibition of FTO reduced hepatic lipogenesis and TG accumulation, implicating a potential strategy for treating NAFLD.

## Results

### Reduced m^6^A methylation is accompanied by elevated FTO level in liver steatosis

**Figure 1:**
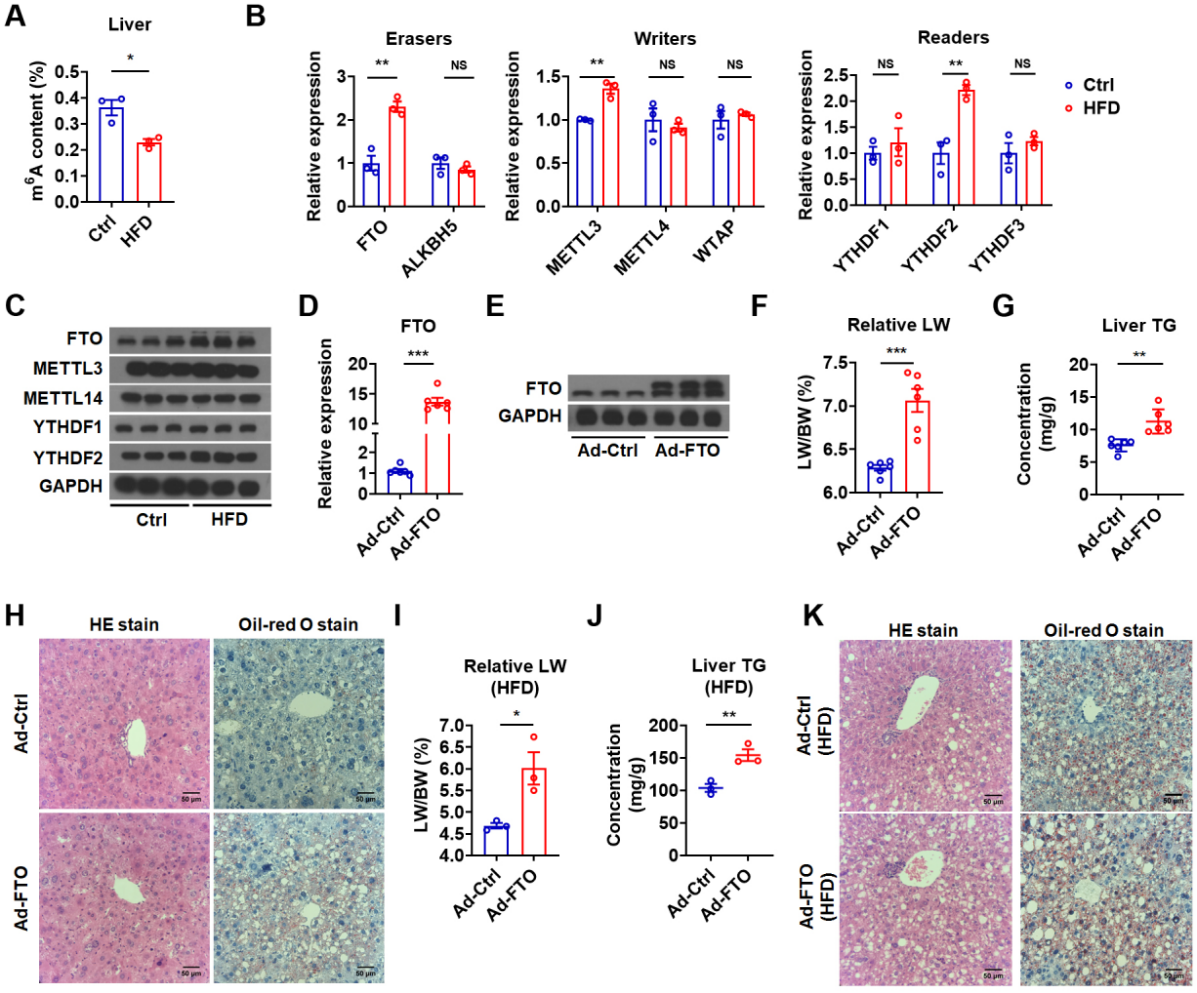
Hepatic FTO is increased in HFD-fed mice and FTO overexpression facilitates hepatic TG accumulation. (**A**) The m^6^A amounts on total RNA in the liver of control and HFD-fed mice (*n *= 3). (**B**) Relative mRNA levels of demethylases (erasers), methyltransferase (writers), and methyl-specific binding proteins (readers) in the liver of control and HFD-fed mice (*n *= 3). (**C**) Protein levels of methyltransferase, demethylases, and methyl-specific binding proteins in the liver of control and HFD-fed mice (*n *= 3). (**D**) Relative mRNA levels of FTO in the liver of mice infected with Ad-Ctrl or Ad-FTO (*n *= 6). (**E**) Protein levels of FTO in the liver of mice infected with Ad-Ctrl or Ad-FTO (*n *= 3). (**F** and **G**) The ratio of liver weight to body weight (LW/BW, **F**) and liver TG contents (**G**) of mice injected with Ad-Ctrl or Ad-FTO (*n *= 6). (**H**) The representative HE and Oil-red O staining images in the liver of mice infected with Ad-Ctrl or Ad-FTO. Scale bar, 50 μm. (**I** and **J**) The LW/BW (**I**) and liver TG contents (**J**) of HFD-fed mice injected with Ad-Ctrl or Ad-FTO (*n *= 3). (**K**) The representative HE and Oil-red O staining images in the liver of HFD-fed mice infected with Ad-Ctrl or Ad-FTO. Scale bar, 50 μm. Data shown are mean ± SEM. *P < 0.05, **P < 0.01, ***P < 0.001. NS denotes not significant.

To investigate whether m^6^A modification is involved in the pathogenesis of hepatic steatosis, we detected the hepatic m^6^A levels in a diet-induced NAFLD mouse model by feeding with high-fat diet (HFD) for 4 months. The overall extent of m^6^A methylation in the liver of these mice was substantially reduced (Figure 1A). Expression analysis of methyltransferases (writers), demethylase (erasers), and methyl binding proteins (readers) revealed that both mRNA and protein levels of hepatic FTO and YTHDF2 were increased. Despite an increase of mRNA, the protein level of METTL3 was unchanged (Figure 1B and C). Considering a decline of overall m^6^A methylation in liver steatosis accompanied by increased FTO expression, we speculated that the demethylase FTO might have a role in hepatic steatosis.

### Hepatic FTO overexpression increases TG accumulation in the liver

To understand whether the increased FTO expression contributes to the development of hepatic steatosis, we overexpressed FTO in the liver of chow diet (CD)-fed mice by tail vein injection of FTO-expressing adenovirus (Ad-FTO) (Figure 1D and E; Supplementary Figure S1A). Two weeks after injection, mice overexpressing FTO displayed increased liver weight (LW) and liver TG content (Figure 1F and G). Hematoxylin and eosin (HE) and Oil-red O staining confirmed that the liver of these mice had histological changes and increased lipid accumulation resembling NAFLD (Figure 1H). There was no significant increase in the weight of adipose tissues (Supplementary Figure S1B and C). These results suggested that hepatic FTO overexpression induced TG accumulation in the liver under normal CD feeding condition. We also overexpressed FTO in the liver of HFD-fed mice (Supplementary Figure S1D). Three weeks after injection, HFD-fed mice overexpressing FTO exhibited heavier LW, higher liver TG content, and more severe hepatic steatosis as compared with HFD-fed control mice (Figure 1I–K). No significant change in the weight of adipose tissues was observed (Supplementary Figure S1E and F). These data indicated that hepatic FTO overexpression could aggravate hepatosteatosis under HFD feeding condition. Thus, a high level of FTO might contribute to TG accumulation in the liver.

### FTO overexpression promotes hepatic TG accumulation by enhancing lipogenesis

**Figure 2:**
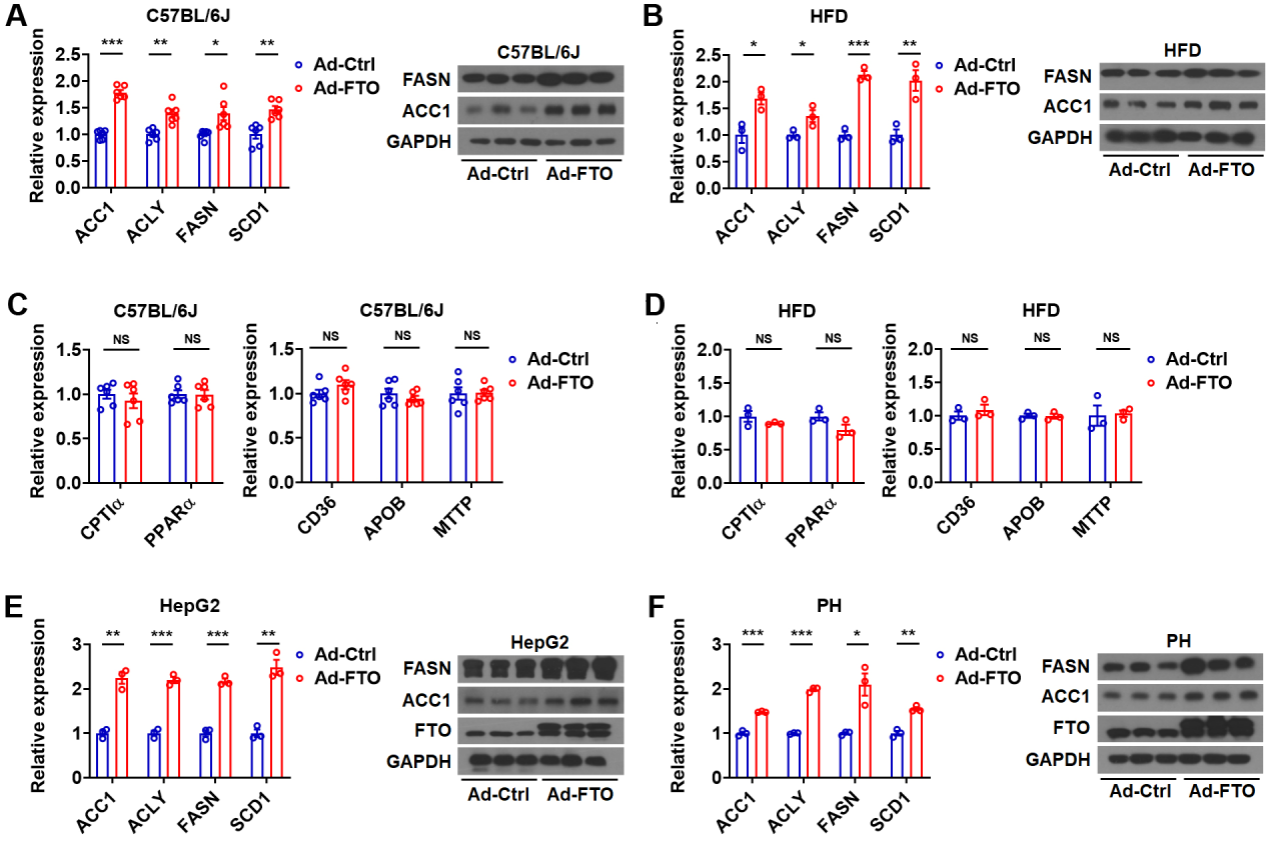
FTO promotes liver TG accumulation by increasing lipogenesis. (**A** and **B**) Relative mRNA levels of lipogenic genes and representative protein levels of FASN and ACC1 in the liver of mice (**A**, *n *= 3) or HFD-fed mice (**B**, *n *= 3) infected with Ad-Ctrl or Ad-FTO. (**C** and **D**) Relative mRNA levels of genes related to fatty acid oxidation (CPTIα and PPARα) and uptake (CD36) and VLDL exportation (APOB and MTTP) in the liver of mice (**C**, *n *= 6) or HFD-fed mice (**D**, *n *= 3) infected with Ad-Ctrl or Ad-FTO. (**E** and **F**) Relative mRNA levels of lipogenic genes and representative protein levels of FASN and ACC1 in HepG2 cells (**E**, *n *= 3) or primary hepatocytes (**F**, *n *= 3) infected with Ad-FTO or Ad-Ctrl. Data shown are mean ± SEM. *P < 0.05, **P < 0.01, ***P < 0.001. NS denotes not significant.

The development of hepatic steatosis is dictated by four aspects: excessive hepatic lipid uptake from systemic and/or portal circulations, increased *de novo* lipid synthesis in hepatocytes, reduced hepatic lipid oxidation/degradation, and decreased lipid export from hepatocytes (Suzuki and Diehl, 2017). To decipher which pathway is responsible for hepatosteatosis after FTO overexpression, key genes involved in lipid metabolism were examined. Indeed, mice overexpressing FTO under either normal CD or HFD feeding condition had higher levels of key lipogenic enzymes in the liver, such as ACLY, ACC1, FASN, and SCD1, compared to the respective control mice (Figure 2A and B). In contrast, no significant change was observed in the mRNA levels of genes involved in fatty acid oxidation (carnitine palmitoyltransferase I alpha (CPTIα) and peroxisome proliferator activated receptor alpha (PPARα)) and uptake (CD36) or VLDL exportation (apolipoprotein B (APOB) and microsomal triglyceride transfer protein (MTTP)) (Figure 2C and D). Consistent with these *in vivo* findings, Ad-FTO infection upregulated the mRNA levels of key lipogenic genes in HepG2 and Hepa1-6 cells *in vitro* (Figure 2E and F; Supplementary Figure S2A). These results suggested that FTO might promote hepatic TG accumulation primarily by increasing lipogenesis.

### Hepatic FTO controls lipogenesis by targeting SREBF1 mRNA

**Figure 3:**
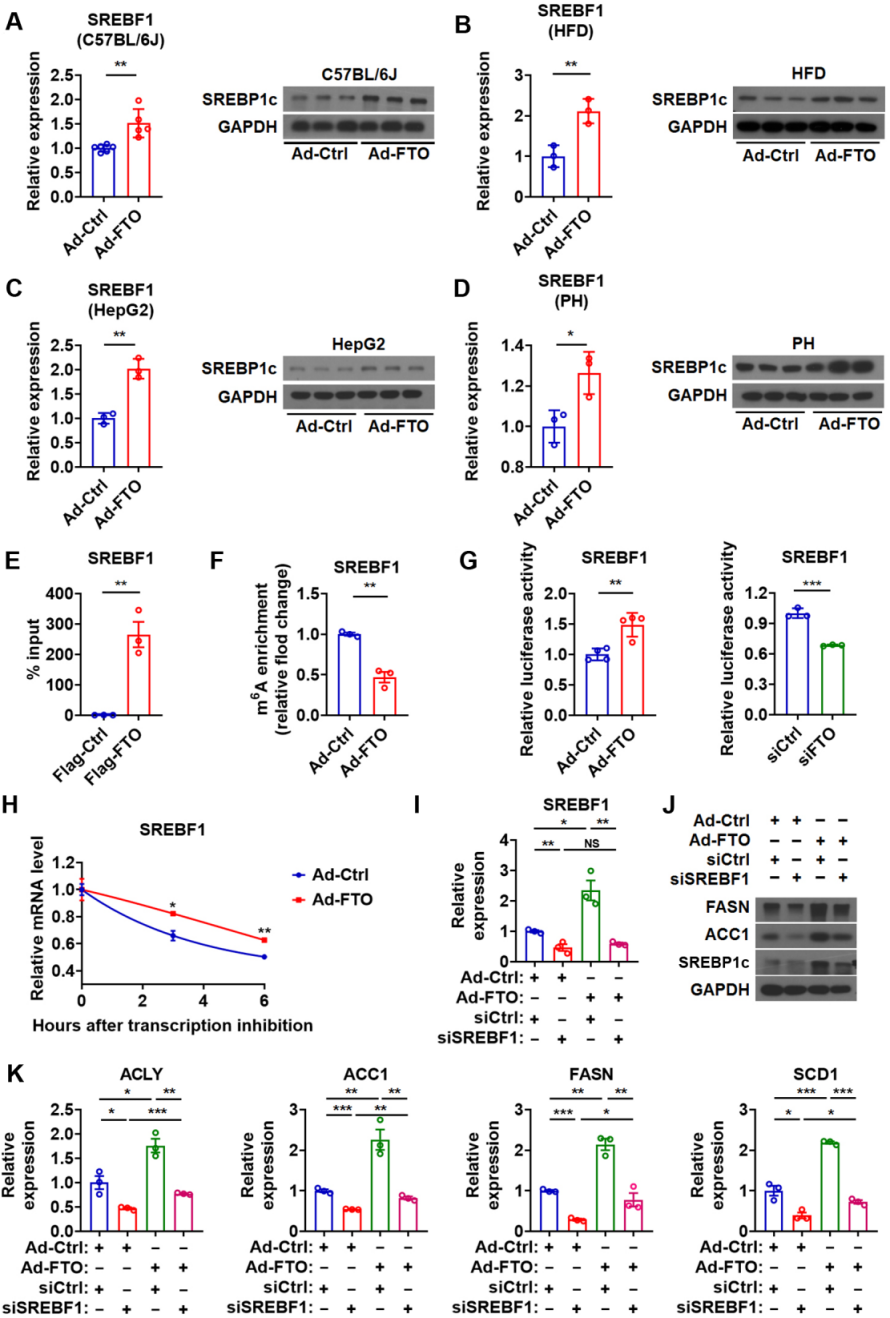
Hepatic FTO controls lipogenesis by targeting SREBF1 mRNA. (**A**) Relative mRNA levels of SREBF1 (n = 6) and protein levels of SREBP1c (*n *= 3) in the liver of Ad-FTO-infected mice. (**B**) Relative mRNA levels of SREBF1 and protein levels of SREBP1c in the liver of HFD-fed mice infected with Ad-FTO (*n *= 3). (**C** and **D**) Relative mRNA levels of SREBF1 and protein levels of SREBP1c in HepG2 cells (**C**) or primary hepatocytes (**D**) infected with Ad-FTO (*n *= 3). (**E**) RIP analysis showing the enrichment of SREBF1 mRNA in the immunoprecipitates containing Flag-FTO in HepG2 cells (*n *= 3). (**F**) MeRIP analysis showing the reduced enrichment of SREBF1 mRNA in m^6^A-containing transcripts derived from the liver of Ad-FTO-infected mice (*n *= 3). (**G**) Luciferase activity of the reporter containing the SREBF1 fragment with putative m^6^A sites after Ad-FTO infection or siFTO transfection in HepG2 cells (*n *= 3). (**H**) The SREBF1 mRNA decay in Hepa1-6 cells after Ad-FTO infection (*n *= 3). (**I**–**K**) Relative mRNA levels of SREBF1 (**I**, *n *= 3), protein levels of ACC1, FASN, and SREBP1c (**J**), and relative mRNA levels of ACLY, ACC1, FASN, and SCD1 (**K**, *n *= 3) in HepG2 cells treated with Ad-FTO and indicated siRNA. Data shown are mean ± SEM. *P < 0.05, **P < 0.01, ***P < 0.001. NS denotes not significant.

Given that SREBP1c controls the transcription of lipogenic genes (Wang et al., 2015c), we tested whether FTO could regulate hepatic lipid metabolism via targeting SREBP1c. We found that both SREBF1 mRNA and SREBP1c protein levels were significantly increased in the liver of mice infected with Ad-FTO under either normal CD or HFD feeding condition, compared to that in control mice under the same feeding condition (Figure 3A and B). In agreement with the above results, the levels of SREBF1 mRNA and SREBP1c protein were increased in cultured hepatocytes *in vitro* after Ad-FTO infection (Figure 3C and D; Supplementary Figure S2B). Moreover, we found that knockdown of FTO by specific small interfering RNA (siRNA) could decrease the expression of SREBF1 and its downstream lipogenic genes at both mRNA and protein levels in cultured hepatocytes (Supplementary Figure S2C and D). These *in vivo* and *in vitro* data suggested that FTO might promote hepatic lipogenesis via SREBP1c.

RNA immunoprecipitation (RIP) showed enrichment of SREBF1 mRNA in HepG2 cells overexpressing FTO, suggesting direct physical bindings between the FTO protein and SREBF1 mRNA (Figure 3E). Consistently, RNA methylation immunoprecipitation (MeRIP) analysis revealed significantly less enrichment of SREBF1 mRNA in m^6^A-containing transcripts derived from the liver of mice infected with Ad-FTO (Figure 3F), suggesting that SREBF1 might be a downstream target of FTO. Indeed, m^6^A methylation of SREBF1 mRNA was significantly upregulated after FTO knockdown in HepG2 cells (Supplementary Figure S2E). We identified three previously unrecognized potential m^6^A modification sites in the 3′UTR region of *SREBF1* gene (Supplementary Figure S2F). Consistently, a reporter containing these sites showed increased activity after Ad-FTO infection and lower activity after FTO knockdown (Figure 3G), suggesting that FTO binding on these three m^6^A sites of SREBF1 increased its expression.

As FTO can regulate the stability of the transcripts of their target genes by demethylating m^6^A sites (Fu et al., 2013; Gu et al., 2020), we tested whether FTO expression changes had any effect on the mRNA stability of SREBF1. As expected, the RNA stability assay showed that overexpression of FTO stabilized SREBF1 mRNA in Hepa1-6 cells (Figure 3H). Collectively, hepatic FTO might promote lipogenesis through enhancing the stability of SREBF1 mRNA via demethylating its m^6^A sites.

To substantiate the pivotal role of SREBP1c in FTO-induced hepatic lipogenesis, we tested whether knockdown of SREBF1 could abolish the effect of FTO (Figure 3I and J; Supplementary Figure S2G). As expected, SREBF1 knockdown greatly attenuated the effect of FTO overexpression on lipogenic genes, confirming the key role of SREBP1c in mediating the effect of FTO on lipogenesis (Figure 3J and K). However, knockdown of SREBF1 was not able to totally abolish the effect of FTO overexpression on lipogenic genes (Figure 3J and K), suggesting that other mechanisms might also be involved.

We also tested whether FTO could regulate the expression of SREBP2, another member of the SREBP family, which is more specific to cholesterogenic gene expression. Ad-FTO infection did not change the mRNA and protein levels of SREBP2 in C57BL/6J mice, HFD-fed mice, HepG2 cells, or primary hepatocytes (Supplementary Figure S3A–D). Moreover, knockdown of FTO could not affect the mRNA and protein levels of SREBP2 in HepG2 cells (Supplementary Figure S3E and F). Furthermore, Ad-FTO treatment did not alter the stability of SREBF2 mRNA (Supplementary Figure S3G). Thus, we concluded that SREBF2 was not under the control of FTO and was not involved in the regulation of hepatic lipid metabolism by FTO.

### Hepatic FTO controls lipogenesis by targeting ChREBP mRNA

**Figure 4:**
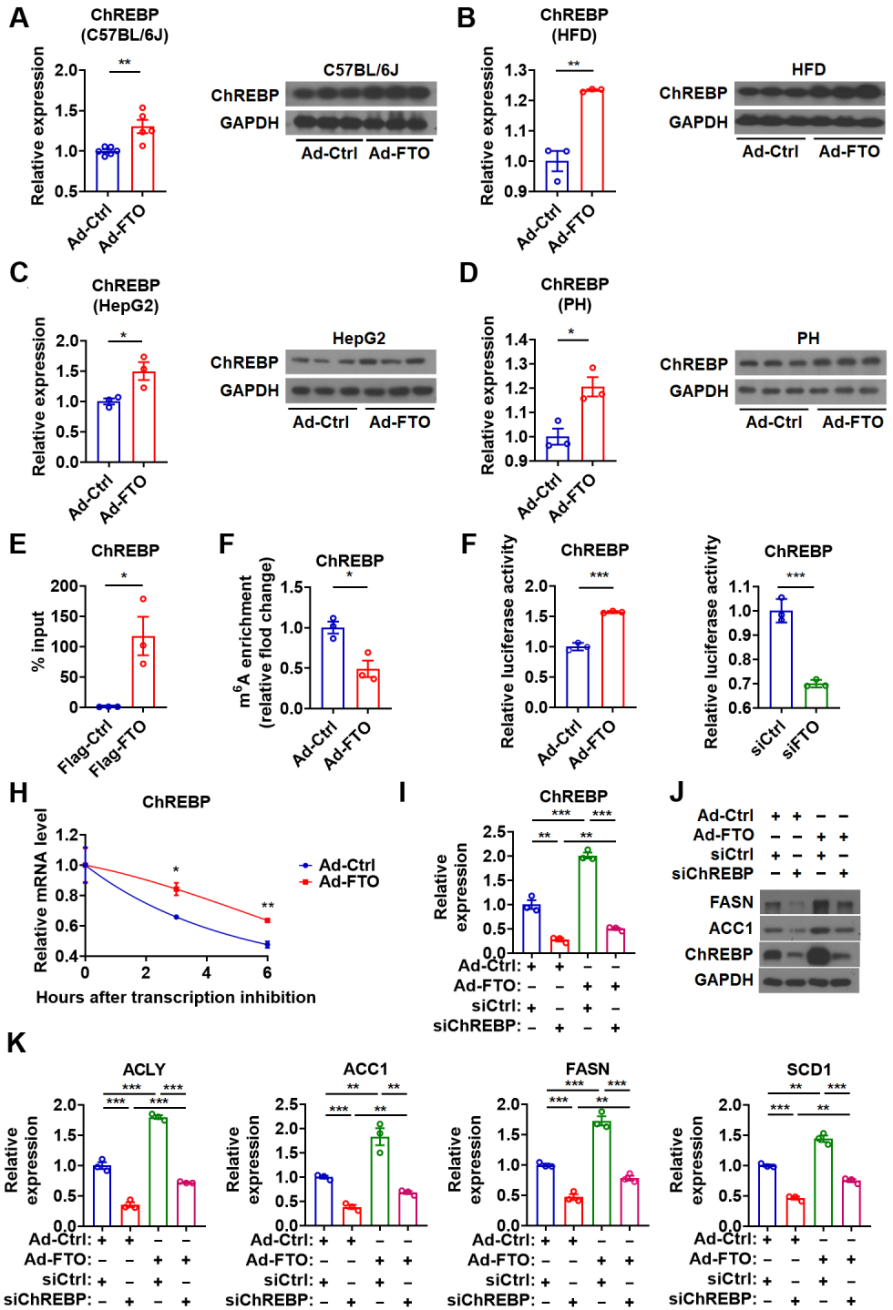
Hepatic FTO controls lipogenesis by targeting ChREBP mRNA. (**A**) Relative mRNA levels of ChREBP (*n *= 6) and protein levels of ChREBP (*n *= 3) in the liver of Ad-FTO-infected mice. (**B**) Relative mRNA and protein levels of ChREBP in the liver of HFD-fed mice infected with Ad-FTO (*n *= 3). (**C** and **D**) Relative mRNA and protein levels of ChREBP in HepG2 cells (**C**) or primary hepatocytes (**D**) infected with Ad-FTO (*n *= 3). (**E**) RIP analysis showing the enrichment of ChREBP mRNA in the immunoprecipitates containing Flag-FTO in HepG2 cells (*n *= 3). (**F**) MeRIP analysis showing the reduced enrichment of ChREBP mRNA in m^6^A-containing transcripts derived from the liver of Ad-FTO-infected mice (*n *= 3). (**G**) Luciferase activity of the reporter containing the ChREBP fragment with putative m^6^A sites after Ad-FTO infection or siFTO transfection in HepG2 cells (*n *= 3). (**H**) The ChREBP mRNA decay in Hepa1-6 cells after Ad-FTO infection (*n *= 3). (**I**–**K**) Relative mRNA levels of ChREBP (**I**, *n *= 3), protein levels of ACC1, FASN, and ChREBP (**J**), and relative mRNA levels of ACC1, ACLY, FASN, and SCD1 (**K**, *n *= 3) in HepG2 cells treated with Ad-FTO and indicated siRNA. Data shown are mean ± SEM. *P < 0.05, **P < 0.01, ***P < 0.001.

It has been suggested that hepatic transcription factors besides SREBP1c, such as ChREBP and LXRα, may also contribute to the induction of lipogenic genes. Interestingly, similar to those observed for SREBP1c, both the mRNA and protein levels of ChREBP were significantly elevated in the liver of mice overexpressing FTO under either normal CD or HFD feeding condition, compared to their controls (Figure 4A and B). Consistently, the mRNA and protein levels of ChREBP were increased in HepG2 and primary hepatocytes after Ad-FTO infection (Figure 4C and D; Supplementary Figure S4A). Furthermore, knockdown of FTO decreased ChREBP at both mRNA and protein levels in HepG2 cells (Supplementary Figure S4B). In contrast, the expression of LXRα was not altered in hepatocytes infected with Ad-FTO both *in vivo* and *in vitro* (Supplementary Figure S4C–E). No significant change in LXRα expression was observed in HepG2 cells after FTO knockdown (Supplementary Figure S4F). Based on these findings, we speculate that SREBP1c and ChREBP, but not LXRα, might be responsible for the regulation of lipogenesis by FTO.

RIP analysis indicated that FTO might demethylate the m^6^A sites of ChREBP mRNA, but not LXRα mRNA, via direct recruitment (Figure 4E; Supplementary Figure S4G). MeRIP analysis suggested that alterations in hepatic FTO expression might be associated with the changes of m^6^A methylation in ChREBP mRNA both *in vivo* and *in vitro* (Figure 4F; Supplementary Figure S4H). Three potential m^6^A modification sites were identified near the stop codon of ChREBP mRNA (Supplementary Figure S4K). As expected, the activity of the reporter containing these sites increased after Ad-FTO infection and decreased after siFTO transfection (Figure 4G). In addition, overexpression of FTO stabilized ChREBP mRNA *in vitro* (Figure 4H). In contrast, the FTO expression changes had no effect on the m^6^A level and stability of LXRα mRNA (Supplementary Figure S4I–L). Together, these findings indicated that FTO might demethylate and stabilize the mRNA of ChREBP, but not LXRα. As ChREBP is critically involved in the biological effect of glucose, we tested whether the glucose levels in culture medium could affect the regulation of ChREBP by FTO. Given that high-glucose culture medium was normally used in the study, we examined ChREBP expression in HepG2 cells after Ad-FTO infection in low-glucose medium. Under a low-glucose culture condition, FTO overexpression could still increase the mRNA and protein levels of ChREBP (Supplementary Figure S4M), suggesting that the regulation of ChREBP by FTO was independent of glucose.

To clarify the crucial role of ChREBP in FTO-induced hepatic lipogenesis, we tested whether downregulation of ChREBP could attenuate the effect of FTO overexpression in cultured hepatocytes. Notably, ChREBP knockdown blocked the effect of FTO on the expression of key lipogenic genes, suggesting a vital role of ChREBP in mediating the effect of FTO on lipogenesis (Figure 4I–K; Supplementary Figure S4N). These data suggested that, besides SREBF1, hepatic FTO might also promote lipogenesis through increasing ChREBP by stabilizing its mRNA via m^6^A demethylation.

In addition, we also tested whether SREBP1c and ChREBP could act synergistically as downstream effectors for FTO in the regulation of lipogenic genes. In line with above findings, knockdown of either SREBF1 or ChREBP totally diminished the effect of Ad-FTO on the mRNA levels of lipogenic genes in HepG2 cells, while a cumulative effect was observed after knockdown of both SREBF1 and ChREBP (Supplementary Figure S5A). Similar results were observed for the effect of either SREBF1 or ChREBP knockdown on ACC1 and FASN protein levels after FTO overexpression. However, cumulative effects were not seen after knockdown of both SREBF1 and ChREBP, indicating a compensatory mechanism (Supplementary Figure S5A). On the other hand, overexpression of either SREBF1 or ChREBP could totally offset the effect of FTO knockdown on the mRNA levels of lipogenic genes in HepG2 cells, while a cumulative effect could be observed after overexpression of both SREBF1 and ChREBP (Supplementary Figure S5B). Although similar results could be observed for the effect of either SREBF1 or ChREBP overexpression on ACC1 and FASN protein levels after FTO knockdown, we were not able to detect a cumulative effect of overexpression of both SREBF1 and ChREBP, indicating the presence of a compensatory mechanism (Supplementary Figure S5B). Given that only cumulative but not synergistic effects were observed (Figures 3I–K and 4I–K; Supplementary Figure S5A and B), we speculated that either ChREBP or SREBF1 played a critical nonredundant role in the regulation of lipogenic genes by FTO. Thus, instead of concluding that one is more important than the other, both ChREBP and SREBF1 could be *bona fide* downstream effectors for FTO and their contribution to FTO-controlled lipogenesis might depend on a physiological or pathological condition or experimental setting.

### Hepatic FTO is involved in the regulation of lipogenesis by insulin

**Figure 5:**
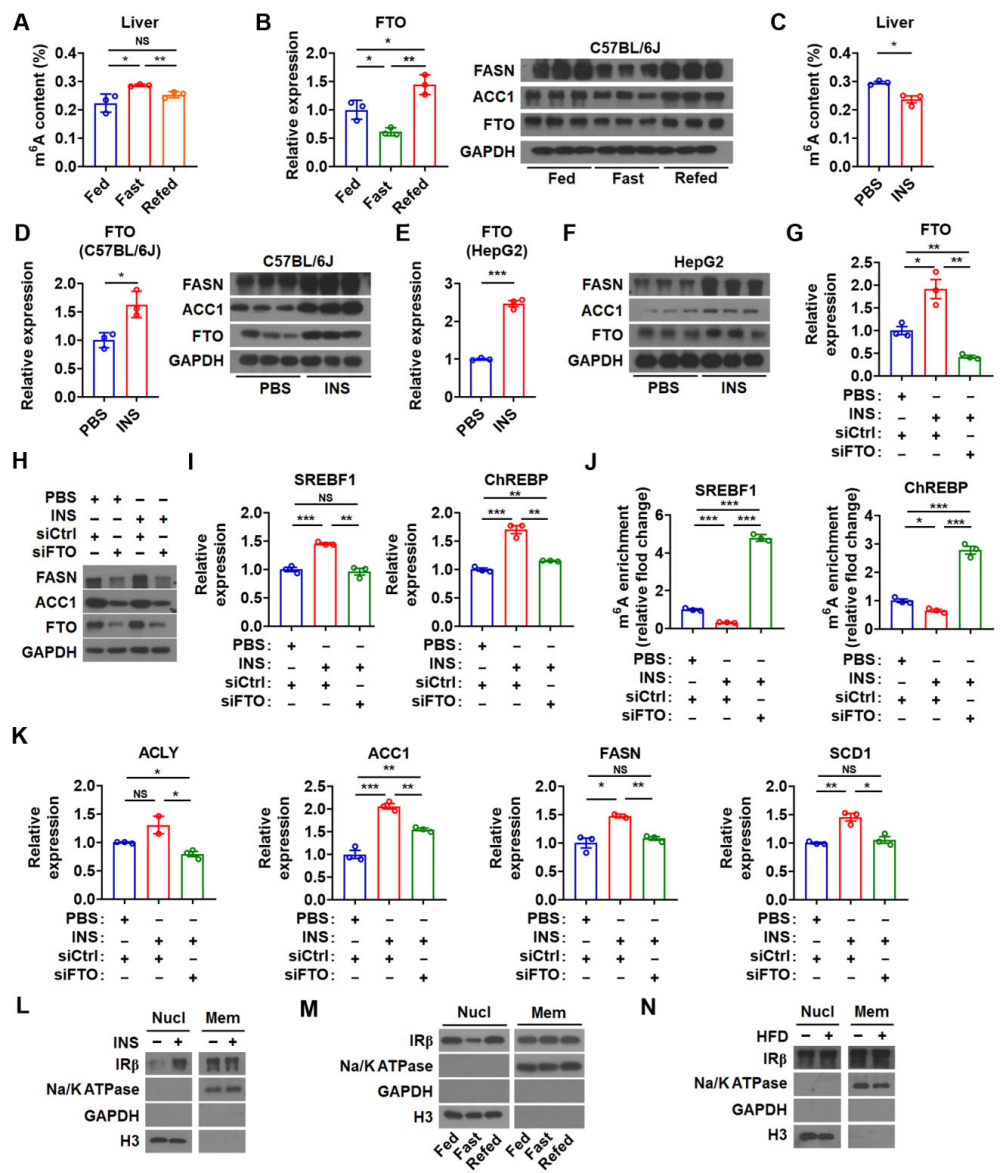
Hepatic FTO is involved in the regulation of lipogenesis by insulin. (**A**) The m^6^A amounts on total RNA in the liver of mice under feeding, fasting, and re-feeding conditions (*n *= 3). (**B**) Relative mRNA levels of FTO (left, *n *= 3) and protein levels of FASN, ACC1, and FTO (right, *n *= 3) in the liver of mice under feeding, fasting, and re-feeding conditions. (**C**) The m^6^A amounts on total RNA in the liver of mice injected with insulin (INS) (*n *= 3). (**D**) Relative mRNA levels of FTO (left) and protein levels of FASN, ACC1, and FTO (right) in the liver of mice injected with INS (*n *= 3). (**E** and **F**) Relative mRNA levels of FTO (**E**, *n *= 3) and protein levels of FASN, ACC1, and FTO (**F**, *n *= 3) in HepG2 cells treated with INS. (**G**–**K**) Relative mRNA levels of FTO (**G**), protein levels of ACC1, FASN, and FTO (**H**), relative mRNA levels of SREBF1 or ChREBP (**I**), the m^6^A enrichment of SREBF1 mRNA or ChREBP mRNA (**J**), and relative mRNA levels of ACLY, ACC1, FASN, and SCD1 (**K**) in HepG2 cells treated with indicated siRNA and INS (*n *= 3). (**L**–**N**) IRβ protein levels in the nuclear fraction (Nucl) and membrane fraction (Mem) from the liver of mice injected with INS (**L**), mice under feeding, fasting, and re-feeding conditions (**M**), or HFD-fed mice (**N**). Data shown are mean ± SEM. *P < 0.05, **P < 0.01, ***P < 0.001. NS denotes not significant.

As the effect of FTO on hepatic lipogenesis resembles that of insulin, a potential link between the insulin signaling pathway and FTO was investigated during the fasting–feeding cycle. After fasting for 6 h, there was a significant increase of m^6^A modification in the liver, accompanied by a decrease of FTO and lipogenic enzymes (Figure 5A and B). In contrast, re-feeding for 6 h led to the opposite effect (Figure 5A and B). Consistently, intraperitoneal insulin injection produced similar effects to that of re-feeding in the liver, i.e. a decrease of m^6^A modification and an increase of FTO and lipogenic enzymes (Figure 5C and D). In line with these data, insulin increased FTO mRNA expression in a dose-dependent manner and enhanced the protein levels of FTO and lipogenic enzymes in HepG2 cells (Figure 5E and F; Supplementary Figure S6A).

To explore whether FTO is critically involved in insulin-stimulated hepatic lipogenesis, we inhibited the expression of FTO by specific siRNA in insulin-treated hepatocytes (Figure 5G and H). Knockdown of FTO attenuated the insulin effect on mRNA expression of both SREBF1 and ChREBP (Figure 5I). MeRIP assay demonstrated a reduction of m^6^A modification on SREBF1 and ChREBP mRNAs after insulin treatment but a dramatic rebound after FTO knockdown (Figure 5J). Accordingly, inhibition of FTO greatly attenuated the effect of insulin on the expression of lipogenic genes (Figure 5H and K). Thus, these findings implied that FTO was under the control of insulin signaling and mediated the action of insulin on hepatic lipogenesis.

Recent studies showed that insulin receptor β (IRβ) could translocate into the nucleus and bind to the promoter region of target genes including FTO in cultured hepatocytes after insulin stimulation (Hancock et al., 2019). Consistently, there was more IRβ protein in the nucleus of hepatocytes from insulin-treated mice (Figure 5L). Moreover, nuclear IRβ protein decreased upon fasting and increased upon re-feeding in hepatocytes (Figure 5M), which was in line with the expression pattern of FTO and the change of m^6^A modification (Figure 5A and B). There was also more nuclear IRβ protein in the hepatocytes of HFD-fed mice (Figure 5N). These data suggested that IRβ might be involved in the regulation of FTO expression by insulin under either physiological or pathological condition.

Additionally, we employed a couple of specific inhibitors for the insulin signaling pathway, targeting different key components downstream of IRβ. Specific inhibitors, including LY294002 for PI3K, MK2206 for AKT, and sb415286 for GSK-3, could not reverse the increase of FTO mRNA induced by insulin (Supplementary Figure S6B and C). These results indicated that intranuclear IRβ, rather than components of insulin signaling downstream of IRβ, played a dominant role in the regulation of FTO expression.

### Pharmaceutical FTO inhibition prevents hepatic TG accumulation

**Figure 6:**
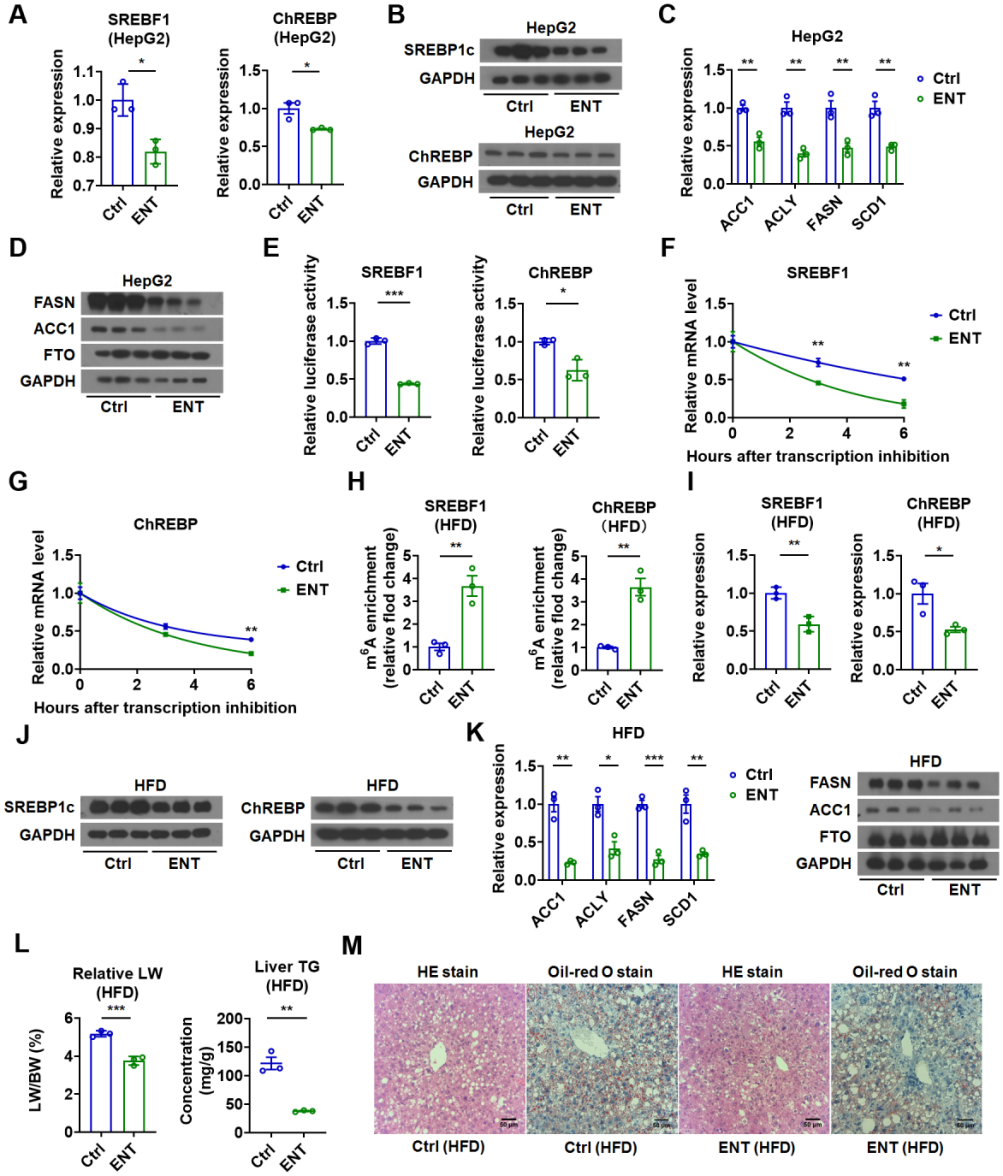
FTO inhibition suppresses lipogenesis and prevents hepatic TG accumulation. (**A**) Relative mRNA levels of SREBF1 and ChREBP in ENT-treated HepG2 cells (*n *= 3). (**B**) Protein levels of SREBP1c and ChREBP in ENT-treated HepG2 cells (*n *= 3). (**C** and **D**) Relative mRNA levels of lipogenic genes and protein levels of FASN, ACC1, and FTO in ENT-treated HepG2 cells (*n *= 3). (**E**) Luciferase activity of the reporter containing the SREBF1 or ChREBP fragment with putative m^6^A sites after ENT treatment in HepG2 cells (*n *= 3). (**F** and **G**) The SREBF1 mRNA or ChREBP mRNA decay in ENT-treated Hepa1-6 cells (*n *= 3). (**H**) MeRIP analysis showing the increased enrichment of SREBF1 and ChREBP mRNAs in m^6^A-containing transcripts derived from the liver of ENT-treated HFD-fed mice (*n *= 3). (**I**) Relative mRNA levels of SREBF1 and ChREBP in the liver of ENT-treated HFD-fed mice (*n *= 3). (**J**) Protein levels of SREBP1c and ChREBP in the liver of ENT-treated HFD-fed mice (*n *= 3). (**K**) Relative mRNA levels of lipogenic genes and representative protein levels of FASN, ACC1, and FTO in the liver of ENT-treated HFD-fed mice (*n *= 3). (**L**) The LW/BW and liver TG contents in ENT-treated HFD-fed mice (*n *= 3). (**M**) The representative HE and Oil-red O staining images of the liver of ENT-treated HFD-fed mice. Scale bar, 50 μm. Data shown are mean ± SEM. *P < 0.05, **P < 0.01, ***P < 0.001.

Given that the expression of key lipogenic enzymes was elevated in the liver of HFD-fed mice (Supplementary Figure S7A) and FTO could positively regulate hepatic lipogenesis, we then employed entacapone (ENT), which is not only a potent inhibitor of FTO but also an FDA-approved drug, to test whether FTO could serve as a potential target to treat hepatosteatosis. Similar to the results observed in FTO knockdown experiments, ENT reduced the expression of both SREBF1 and ChREBP (but not LXRα) as well as key lipogenic enzymes in hepatocytes (Figure 6A–D; Supplementary Figure S7B–D). The activity of the reporter containing the SREBF1 or ChREBP fragment with predicted m^6^A sites was reduced after FTO inhibition by ENT treatment (Figure 6E). Accordingly, RNA stability assay also showed that FTO inhibition by ENT destabilized both SREBF1 and ChREBP (but not LXRα) mRNAs in hepatocytes (Figure 6F and G; Supplementary Figure S7E).

To validate the specificity of ENT on FTO inhibition and consequent lipogenic changes, we examined the levels of SREBF1, ChREBP, and lipogenic enzymes after ENT treatment of HepG2 cells overexpressing FTO. ENT treatment greatly attenuated the effect of FTO overexpression on lipogenic genes (Supplementary Figure S8A). Notably, two-way analysis of variance (ANOVA) of the quantitative polymerase chain reaction (qPCR) results revealed a significant FTO overexpression-by-ENT treatment interaction concerning the mRNA expression of these lipogenic genes, indicating that the ENT treatment-related differences in the mRNA expression of these genes might depend on FTO overexpression (Supplementary Table S1). On the other hand, the levels of these lipogenic genes after ENT treatment were examined in HepG2 cells with FTO knockdown. The effect of ENT on the expression of these genes was totally abolished after FTO knockdown (Supplementary Figure S8B), further supporting the notion that FTO was required for the ENT action on these lipogenic genes. These conclusions were further substantiated by two-way ANOVA of the qPCR results revealing a significant FTO knockdown-by-ENT treatment interaction concerning the mRNA levels of these lipogenic genes (Supplementary Table S1). Together, these results suggested that the ENT effect on FTO was specific and the regulatory effect of ENT on the mRNA expression of lipogenic genes was attributed to its inhibitory effect on FTO.

We then treated HFD-fed mice with ENT to examine whether ENT could reduce obesity-associated hepatic TG accumulation. As expected, MeRIP analysis revealed that ENT treatment increased m^6^A modification of SREBF1 and ChREBP mRNAs in the liver of HFD-fed mice (Figure 6H). The expression of SREBF1 and ChREBP (but not LXRα and SREBF2) and downstream lipogenic genes were all reduced in the liver of HFD-fed mice after ENT treatment (Figure 6I–K; Supplementary Figure S9A–C). Remarkably, ENT led to reduced LW and hepatic TG content (Figure 6L) and significant improvement in hepatic steatosis in HFD-fed mice, as shown by HE and Oil-red O staining (Figure 6M). These findings implied that suppression of hepatic lipogenesis by inhibiting FTO could serve as a potential strategy for treating fatty liver.

## Discussion

It has been accepted that increased hepatic *de novo* lipogenesis contributes greatly to the development of steatosis in NAFLD. Although the mechanism responsible for this increase in hepatic lipogenesis is unclear, selective insulin resistance has long been observed in fatty liver, which is manifested by insulin resistance to the suppression of hepatic glucose production but preserves insulin sensitivity in the SREBP1c pathway that stimulates fatty acid synthesis. It has been suggested that hyperinsulinemia activates SREBP1c to promote hepatic TG accumulation in NAFLD patients, though the detailed mechanism remains unclear. FTO is an obesity-susceptible gene identified through genome-wide association studies (Scuteri et al., 2007). FTO levels were found to be increased in the fatty liver of rodents and patients with NAFLD (Guo et al., 2013; Lim et al., 2016). Although FTO has been implicated in the regulation of hepatic lipogenesis, the physiological or pathophysiological role of FTO in hepatic lipogenesis is not fully understood.

Here, we first observed an increase of FTO in the liver of HFD-fed mice. Then, a causal link between FTO and steatosis was established by *in vivo* FTO overexpression. Next, we found that FTO induced the expression of genes involved in lipogenesis but not in lipoprotein transport or fatty acid β-oxidation. Following the lead of lipogenesis, we traced back to an increase of its upstream regulators, i.e. SREBP1c and ChREBP, whose mRNAs were direct substrates of FTO. FTO bound to and demethylated m^6^A in SREBF1 and ChREBP mRNAs, enhancing their stability. Since SREBP1c functions in insulin-mediated lipogenesis, we further established that FTO was regulated by insulin and also mediated the lipogenic effect of insulin. Although our data showed that either ChREBP or SREBF1 had a nonredundant role in the regulation of lipogenic genes by FTO, given that only cumulative but not synergistic actions were observed for ChREBP and SREBF1 in hepatocytes in response to FTO, we speculated that these two transcription factors were *bona fide* downstream effectors for FTO and their contribution to the FTO effect on lipogenesis might depend on physiological and pathological conditions. Here, we deciphered an elegant insulin/FTO/SREBP1c/ChREBP/lipogenesis signaling pathway, consolidating FTO as an important contributor to fatty liver. Through its demethylase activity, FTO added a layer of dynamic gene regulation in hepatic TG metabolism.

A recent study demonstrated that YTH domain-containing 2, an m^6^A reader, could bind to the mRNA of lipogenic genes, decrease their mRNA stability, and inhibit lipogenesis (Zhou et al., 2021). In light of these findings, m^6^A methylation seems to play an important role in the regulation of hepatic lipid metabolism. In our study, we observed an increase of FTO in the fatty liver of HFD mice, consistent with previous findings in the fatty liver of rats and patients with nonalcoholic steatohepatitis (Guo et al., 2013; Lim et al., 2016), and a corresponding decrease in m^6^A methylation. We did not find any change in other m^6^A writer or eraser proteins examined in this study. In contrast, a decrease of FTO and an increase of the writer METTL3 has been reported in the liver of *db/db* mice, accompanied by an increase of global m^6^A methylation (Zhou et al., 2021). The mechanism underlying this discrepancy is unclear, but it is conceivable that the consequence of m^6^A methylation is complex and context- and gene-dependent. Results obtained from different models should be interpreted with caution.

As the list of m^6^A target mRNAs is growing, the role of m^6^A modification in cellular, developmental, and disease processes has been attracting extensive attention (Roundtree et al., 2017). Emerging evidence also suggests the therapeutic potential of targeting m^6^A regulators in diseases. Although FTO has long been linked to obesity, an increasing interest in FTO in metabolic regulation has recently emerged (Yang et al., 2022). Recent studies from various species or in different metabolic tissues have highlighted the crucial role of FTO in regulating many aspects of lipid homeostasis, including lipogenesis, lipolysis, lipid transportation, lipid uptake, fatty acid oxidation, and adipogenesis, by targeting many genes (Mo et al., 2017; Wu et al., 2017; Chen et al., 2018; Kang et al., 2018; Wei et al., 2022). Nevertheless, relatively few genes, such as CES2, PPARγ, and PLIN5 (Mo et al., 2017; Takemoto et al., 2021; Wei et al., 2021), have been established as direct substrates of FTO. To what extent are these identified targets responsible for the FTO action on lipid metabolism and how they coordinate FTO-controlled lipid metabolism in a context- and tissue-specific manner are not fully understood and require further study in the future.

Structure-based virtual screening identified ENT, an inhibitor of catechol-O-methyltransferase, as a potent FTO chemical inhibitor (Peng et al., 2019). Previous study in the liver mainly focused on ENT effects on suppressing hepatic gluconeogenesis and improving glucose tolerance. In our study, hepatic FTO inhibition with ENT significantly ameliorated hepatosteatosis *in vivo*, raising FTO as a promising target for treating diet-induced hepatic metabolic diseases. So far, there is still huge unmet clinical need in the field of NAFLD, without FDA-approved medication available. It would be interesting to develop more FTO inhibitors and test their efficacies in treating NAFLD.

In summary, our study established a functional role of FTO during the fasting–feeding cycle and in the pathogenesis of NAFLD, which responded to insulin and promoted hepatic lipogenesis by stabilizing SREBF1 and ChREBP mRNAs. Our findings provide new insights into the role of m^6^A modification in the regulation of hepatic lipid metabolism in response to hormones and nutrients. Hepatic FTO targeting strategy deserves to be further explored for its potential in treating NAFLD in the future.

## Materials and methods

### Animal experiments

Male C57BL/6J mice purchased from Shanghai Laboratory Animal Company were kept at 22°C–23°C and 35% ± 5% humidity with a 12-h dark/light cycle, in the specific pathogen-free facility of Shanghai Institute of Nutrition and Health (SINH), Chinese Academy of Sciences (CAS). To induce hepatic steatosis, 4-week-old mice were fed with HFD (Research Diets, D12492) for 16 weeks. Overexpression of FTO in the liver of 8-week-old mice was achieved by tail vein injection of adenoviral FTO (Ad-FTO) or control adenovirus (Ad-Ctrl) (5 × 10^7^ pfu/g). Two weeks after injection, mice were sacrificed and tissues were collected. HFD-fed mice were also injected with Ad-FTO or Ad-Ctrl (5 × 10^7^ pfu/g). After 3 weeks, mice were sacrificed and tissue samples were collected. For pharmaceutical inhibition of FTO, HFD-fed mice were injected daily with ENT (200 mg/kg, CSNpharm, CSN17052) or equivalent volume of solvent for 2 weeks. Then, mice were sacrificed, and tissues were collected. All experiments were approved by the Animal Care and Use Committee of SINH, CAS (2016-AN-1, SIBS-2019-YH-1).

### Liver histology

The fresh liver tissues were fixed in 4% paraformaldehyde for 24 h. Paraffin-embedded samples were sectioned at 5 μm and sequentially stained with HE. For Oil-red O staining, liver tissues were first fixed with sequential 10% and 20% sucrose equilibration, each for 12 h at 4°C, and then cryoembedded in optimal cutting temperature medium. The 10-μm cryostat sections were prepared and stained with Oil-red O for lipids and hematoxylin for nuclei. Finally, HE and Oil-red O staining images were taken using a light microscope (Olympus).

### Mouse primary hepatocyte isolation and culture

Mouse primary hepatocyte isolation and culture was performed as previously described (Liu et al., 2014b). Briefly, 8-week-old mice were anaesthetized with sodium pentobarbital (30 mg/kg i.p.). The portal vein was cannulated under aseptic conditions and the liver was first perfused with phosphate-buffered saline containing 5 mM EGTA and 1% HEPES, and then perfused with digestion buffer containing 0.05% collagenase type I solution. The digestion was quenched by Dulbecco's modified Eagle's medium (DMEM) containing 10% fetal bovine serum (FBS). The isolated hepatocytes were filtered through a 70-mm cell strainer, centrifuged, and washed. Finally, hepatocytes were re-suspended with DMEM (10% FBS) and cultured overnight at 1 × 10^6^ cells per well in 6-well plates coated with rat-tail collagen type I.

### Cell culture and treatment

Hepa1-6 and HepG2 cells were maintained in high-glucose DMEM (Gibco, C11995500BT), unless otherwise indicated, with 10% heat-inactivated FBS. To test whether FTO is involved in the regulation of ChREBP under low-glucose condition, HepG2 cells were cultured in low-glucose DMEM (Gibco, 31600034) with 10% heat-inactivated FBS as indicated. Transfection of siRNA (20 nM per well) was conducted using Lipofectamine RNAiMAX (Invitrogen, 13778150). The siRNA sequences are listed in Supplementary Table S2. ENT (50 μM) was added when the medium was changed after FTO overexpression or knockdown. To investigate the effect of components of insulin signaling downstream of IRβ on FTO mRNA expression, the cells were pretreated with ly294002 (PI3K inhibitor, 10 μM), sb415286 (GSK-3 inhibitor, 10 μM), or mk2206 (Akt inhibitor, 3 μM) for 2 h before addition of insulin (100 nM).

### Plasmid construction and adenovirus recombination

The full-length open reading frames (ORFs) of human *FTO, SREBF1*, and *MLXIPL* (ChREBP) genes were amplified from HepG2 cDNA. The ORF of *FTO* was cloned into a flag-tagged pcDNA3.1 mammalian expression vector (Invitrogen, V790-20). The ORFs of *SREBF1* and *MLXIPL* were cloned into a pLVX-puro lentiviral expression vector (Clontech, 632164). m^6^A site prediction was conducted by using a sequence-based m^6^A modification site predictor from the website (http://www.cuilab.cn/sramp). Then, the m^6^A site-containing fragment was amplified from HepG2 cDNA and cloned into the pRL-TK vector for dual-luciferase reporter assay. Recombinant adenovirus for FTO overexpression was generated using the AdEasy Adenoviral Vector System (Stratagene, RRID:Addgene_18703) in 293A cells. For *in vivo* FTO overexpression, viruses were used at 5 × 10^7^ pfu/g via tail vein injection. For *in vitro* transfection using cell lines or primary hepatocytes, viruses were used at the multiplicity of infection of 100. Primers are listed in Supplementary Table S3.

### m^6^A RNA methylation quantification

The m^6^A methylation of RNA was quantified by m^6^A RNA methylation Quantification Kit (colorimetric, ab185912). Briefly, total RNA was bound to the strip wells through RNA high binding solution, followed by adding capture antibody solution and detection antibody solution. The absorbance at 450 nm wavelength was read by a spectrophotometer for colorimetric quantification. The content of m^6^A was directly proportional to the measured optical density value.

### mRNA stability assay

Hepa1-6 cells were placed in 12-well plates and cultured overnight. After pretreatment with adenovirus or ENT for 48 h, cells were treated with 5 μg/ml actinomycin D (act-D, Sigma, a9415) to inhibit RNA transcription. Cells were collected at 0, 3, and 6 h after adding act-D. The mRNA expression for each group at the indicated time was calculated and normalized by 18S RNA.

### Luciferase assay

HepG2 cells were placed in 48-well plates and cultured overnight. Cells were transfected with adenovirus or siRNA. After 24 h, each well was co-transfected with 0.24 μg PGL3 basic plasmid and 0.02 μg pRL TK plasmid and cultured for another 24 h. Then, the dual luciferase activity was measured by a luminometer with the Dual-Luciferase Reporter Assay System (Promega, E1960, RRID:SCR_020536) according to the manufacturer's protocol. The results were represented as the ratio of firefly and Renilla Luciferase activity.

### m^6^A MeRIP

m^6^A MeRIP was measured by using Magna MeRIP m^6^A Kit (Millipore, 17-10499) following the manufacturer's instructions. Briefly, 30 μg RNA was saved as input for qPCR. The m^6^A antibody (10 μg) and magnetic beads (50 μl) were incubated and rotated in IP buffer for 30 min at room temperature. After washing, 300 μg RNA was mixed with beads in IP buffer containing RNase inhibitor, incubated, and rotated overnight at 4°C. Finally, the enriched RNA with m^6^A was eluted, using RNeasy Kit (Qiagen, 74106). The m^6^A site methylation levels of indicated genes were analyzed by qPCR.

### RIP

RIP experiments were performed with the Magna RIP RNA Binding Protein Immunoprecipitation Kit (Millipore, 17-701). Briefly, flag-tagged FTO (Flag-FTO) protein was first expressed by plasmid transfection in HepG2 cells. HepG2 cells were lysed with RIP lysis buffer. Cell lysates were immunoprecipitated with anti-flag antibody and incubated with protein A/G magnetic beads overnight at 4°C. The precipitated RNA was purified, reverse-transcribed into cDNA, and finally quantified by qPCR.

### Data analysis

The experimental data were analyzed and plotted using Excel and Graphpad 7.0 software (GraphPad Software). All data were represented as mean ± standard error of the mean (SEM) of at least three independent experiments. Statistical significance was assessed by an unpaired Student's *t*-test unless otherwise indicated. To evaluate the interaction between two independent variables, two-way ANOVA analysis with Tukey's multiple comparison test was used as indicated. A *P*-value of <0.05 was considered to be of statistical significance.

## Supplementary Material

mjac061_Supplemental_File
