# Supplementary material for: Aberrant elevation of FTO levels promotes liver steatosis by decreasing the m6A methylation and increasing the stability of SREBF1 and ChREBP mRNAs
Source: J Mol Cell Biol. 2022 Nov 10;14(9):mjac061. doi: 10.1093/jmcb/mjac061 (PMC9951264; doi:10.1093/jmcb/mjac061)
Supplement: mjac061_Supplemental_File [file mjac061_supplemental_file.pdf]

Supplementary material

Aberrant elevation of FTO levels promotes liver steatosis by decreasing the m^6A methylation and increasing the stability of SREBF1 and ChREBP mRNAs

Zhili Tang[1,2], Chao Sun[1], Ying Yan[1], Zhoumin Niu[1], Yuying Li[1], Xi Xu[3], Jing Zhang[3], Yuting Wu[1], Yan Li[4], Li Wang[4], Cheng Hu[5], Zhuoyang Li[1,*], Jingjing Jiang[3,*], and Hao Ying [1,2,6,*]

[1] CAS Key Laboratory of Nutrition, Metabolism and Food Safety, Shanghai Institute of Nutrition and Health, University of Chinese Academy of Sciences, Chinese Academy of Sciences, and Shanghai Jiao Tong University Affiliated Sixth People's Hospital, Shanghai 200031, China

[2] Innovation Center for Intervention of Chronic Disease and Promotion of Health, Shanghai 200031, China

[3] Department of Endocrinology and Metabolism, Zhongshan Hospital, Fudan University, Shanghai 200031, China

[4] State Key Laboratory of Food Science and Technology, School of Food Science and Technology, Jiangnan University, Wuxi 214122, China

[5] Shanghai Diabetes Institute, Shanghai Key Laboratory of Diabetes Mellitus, Shanghai Clinical Centre for Diabetes, Shanghai Jiao Tong University Affiliated Sixth People's Hospital, Shanghai 200233, China

[6] Key Laboratory of Food Safety Risk Assessment, Ministry of Health, Beijing 100021, China

* Correspondence to: Zhuoyang Li, E-mail: lizhuoyang2020@sibs.ac.cn; Jingjing Jiang, E-mail: jiang.jingjing@zs-hospital.sh.cn; Hao Ying, E-mail: yinghao@sibs.ac.cn

Supplementary Figures

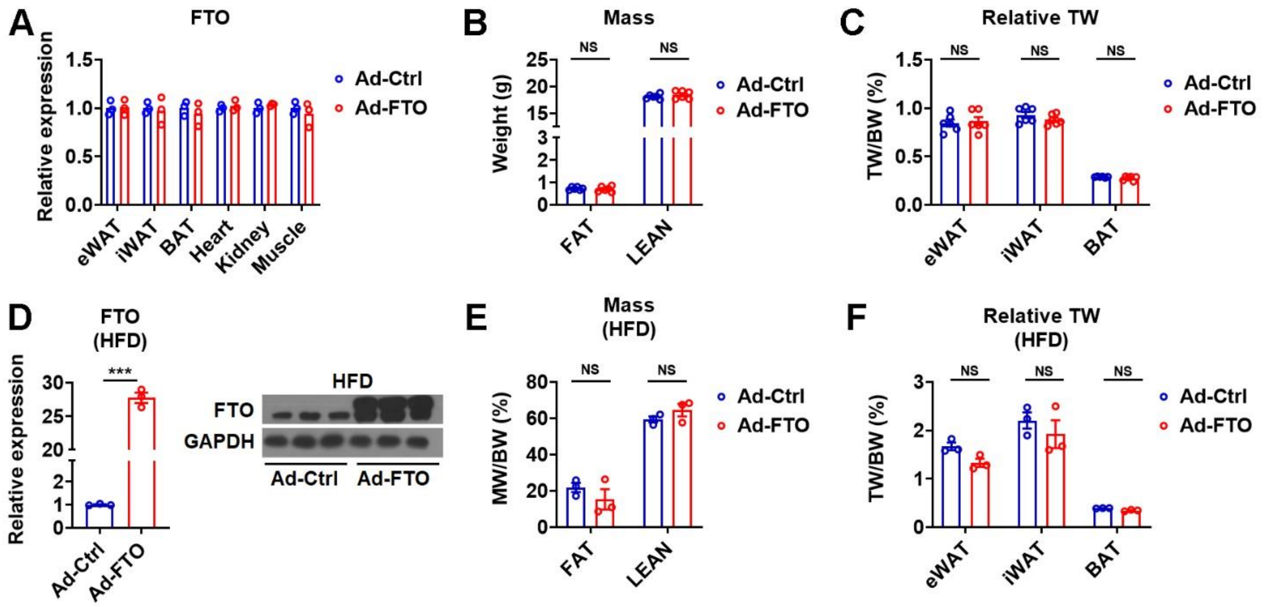

Supplementary Figure S1. Other tissues are not affected by hepatic FTO overexpression.

(**A**) Relative mRNA levels of FTO in other tissues of mice infected with Ad-Ctrl or Ad-FTO (n=3). (**B**) The fat mass and lean mass of mice infected with Ad-Ctrl or Ad-FTO (n=6). (**C**) The ratio of adipose tissue weight to body weight (TW/BW) of mice infected with Ad-Ctrl or Ad-FTO (n=6). (**D**) Relative mRNA levels of FTO and FTO protein levels in the liver of HFD-fed mice infected with Ad-Ctrl or Ad-FTO (n=3). (**E**) The ratio of fat mass and lean mass to body weight (MW/BW) of HFD-fed mice infected with Ad-Ctrl or Ad-FTO (n=3). (**F**) The ratio of adipose tissue weight to body weight (TW/BW) of HFD-fed mice infected with Ad-Ctrl or Ad-FTO (n=3). Means ±SEM are shown. *p<0.05, **p<0.01, ***p<0.001. NS denotes not significant.

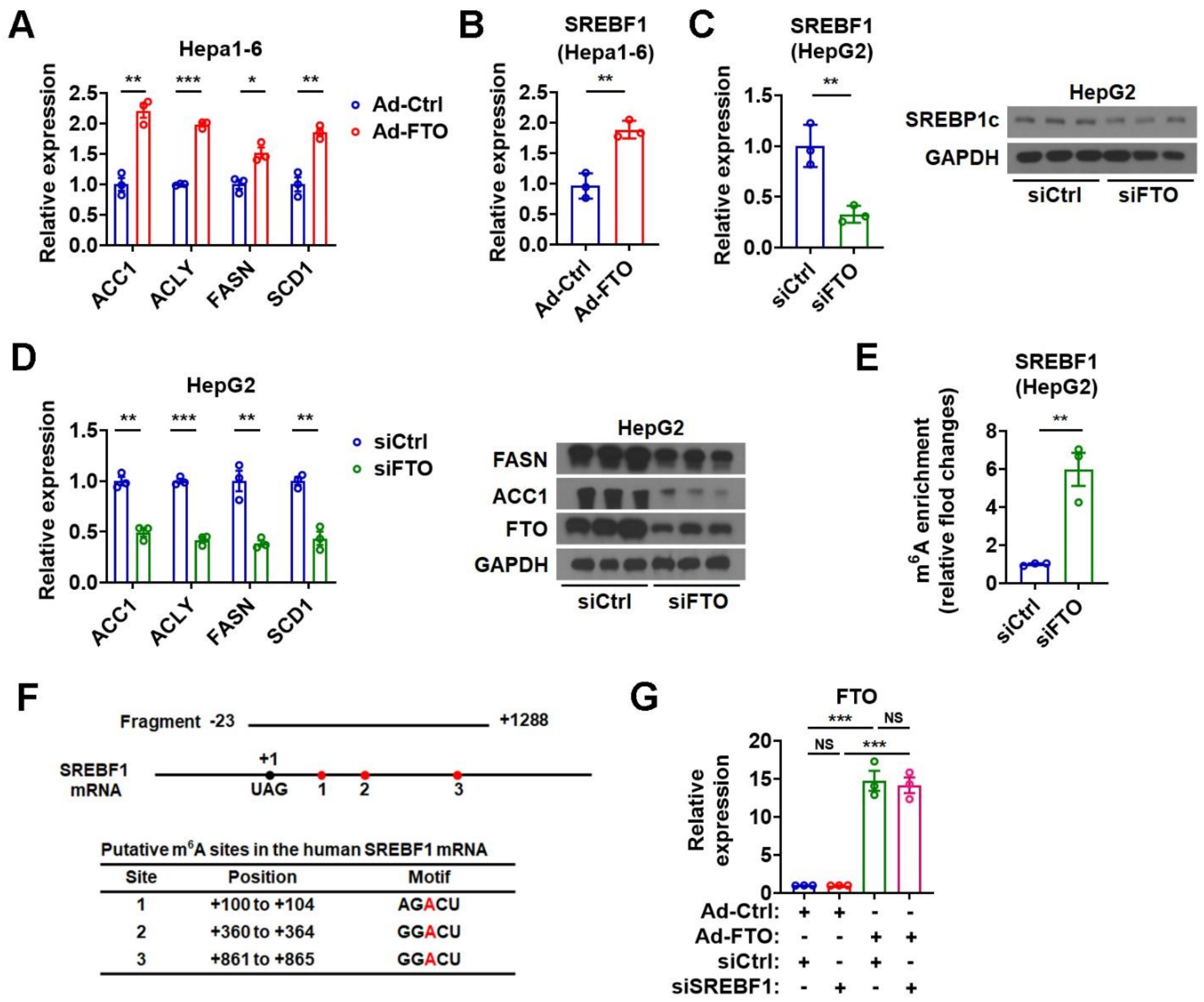

Supplementary Figure S2. FTO regulates SREBF1 expression in cultured hepatocytes.

(**A**) Relative mRNA levels of lipogenic genes in Hepa1-6 cells infected with Ad-Ctrl or Ad-FTO (n=3). (**B**) Relative mRNA levels of SREBF1 in Hepa1-6 cells infected with Ad-Ctrl or Ad-FTO (n=3). (**C**) Relative mRNA levels of SREBF1 and protein levels of SREBP1c in HepG2 cells transfected with indicated siRNA (n=3). (**D**) Relative mRNA levels of lipogenic genes (left) and protein levels of FASN, ACC1, and FTO (right) in HepG2 cells transfected with indicated siRNA (n=3). (**E**) MeRIP analysis showing the increased enrichment of SREBF1 mRNA in m^{6}A-containing transcripts derived from HepG2 cells transfected with siFTO (n=3). (**F**) Putative m^{6}A sites in human SREBF1 mRNA and the fragment used for the cloning of the reporter. (**G**) Relative mRNA levels of FTO in HepG2 cells treated with Ad-FTO and siSREBF1 (n=3). Means ±SEM are shown. *p<0.05, **p<0.01, ***p<0.001. NS denotes not significant.

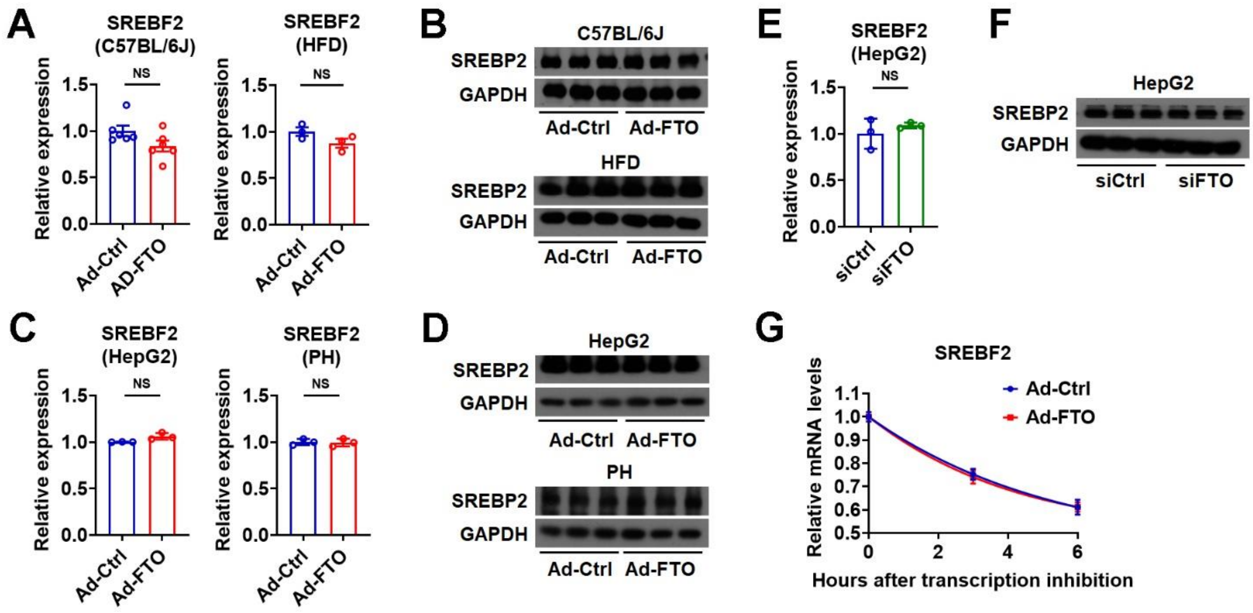

Supplementary Figure S3. Hepatic FTO does not regulate the expression of SREBF2.

(**A**) Relative mRNA levels of SREBF2 (n=6) in the liver of Ad-FTO-infected C57BL/6J mice (n=6) and HFD-fed mice (n=3). (**B**) Protein levels of SREBP2 in the liver of Ad-FTO-infected C57BL/6J mice (n=6) and HFD-fed mice (n=3). (**C** and **D**) Relative mRNA levels of SREBF2 (**C**) and protein levels of SREBP2 (**D**) in HepG2 cells or primary hepatocytes (PH) infected with Ad-FTO (n=3). (**E** and **F**) Relative mRNA levels (**E**) and protein levels (**F**) of SREBF2 in HepG2 cells transfected with indicated siRNA (n=3). (**G**) The SREBF2 mRNA decay in Hepa1-6 cells after Ad-FTO infection (n=3). Means±SEM are shown. *p<0.05, **p<0.01, ***p<0.001. NS denotes not significant.

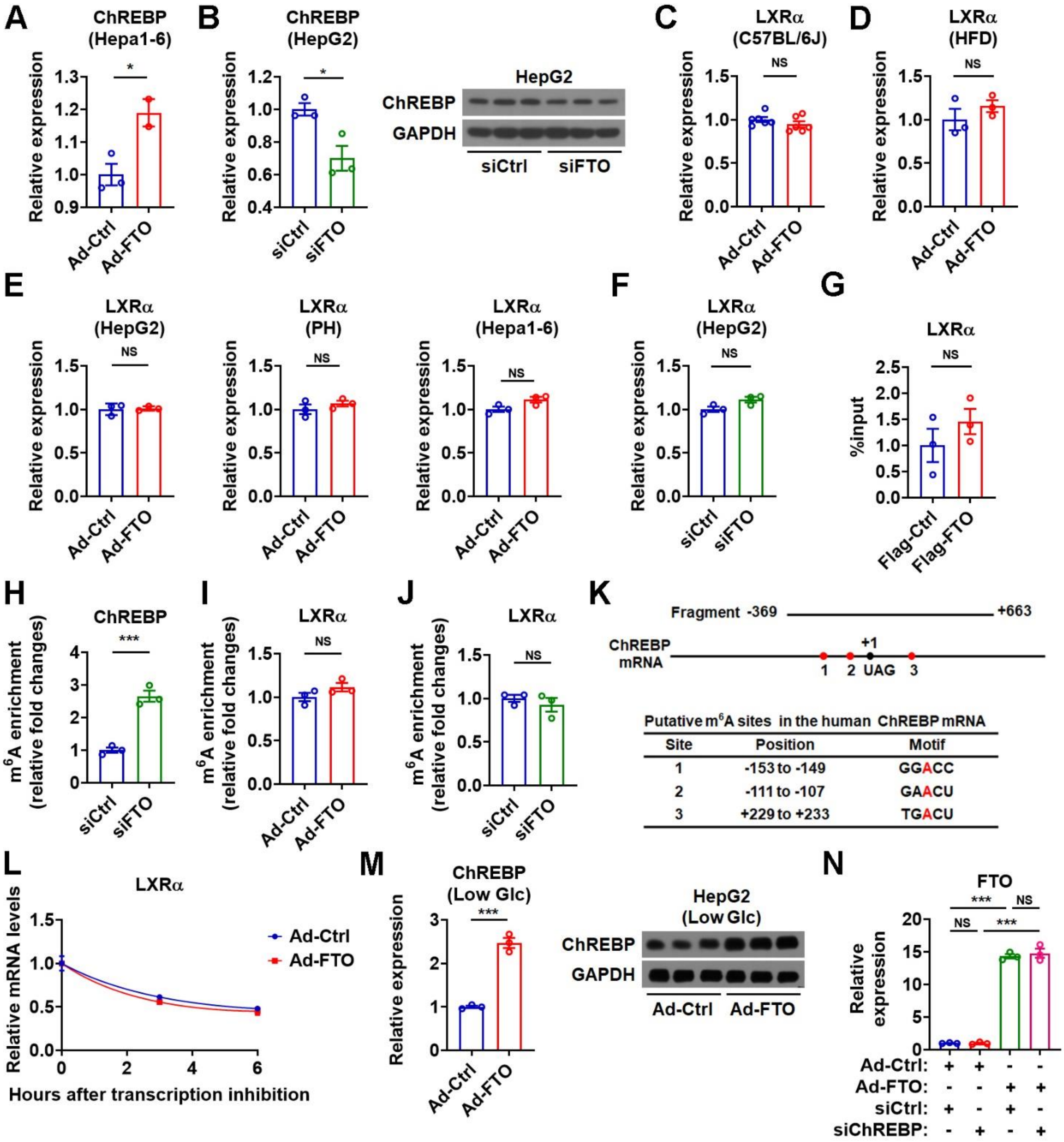

Supplementary Figure S4. FTO regulates the expression of ChREBP but not LXRα in cultured hepatocytes.

(**A**) Relative mRNA levels of ChREBP in Hepa1-6 cells infected with Ad-Ctrl or Ad-FTO (n=3). (**B**) Relative mRNA levels of ChREBP and protein levels of ChREBP in HepG2 cells transfected with indicated siRNA (n=3). (**C**) Relative mRNA levels of LXRα in the liver of Ad-Ctrl or Ad-FTO-infected mice (n=3). (**D**) Relative mRNA levels of LXRα in the liver of HFD-fed mice infected with Ad-Ctrl or Ad-FTO (n=3). (**E**) Relative mRNA levels of LXRα in HepG2 cells, primary hepatocytes, and Hepa1-6 cells infected with Ad-Ctrl or Ad-FTO (n=3). (**F**) Relative mRNA levels of LXRα in HepG2 cells transfected with indicated siRNA (n=3). (**G**) RIP analysis showing the enrichment of LXRα mRNA in HepG2 cells transfected with Flag-FTO or Flag-Ctrl (n=3). (**H**) MeRIP analysis showing the increased enrichment of ChREBP mRNA in m^6A-containing transcripts derived from HepG2 cells transfected with siFTO (n=3). (**I**) MeRIP analysis showing the

enrichment of LXRα mRNA in m^{6}A-containing transcripts derived from the liver of mice infected with Ad-Ctrl or Ad-FTO (n=3). (**J**) MeRIP analysis showing the unaltered enrichment of LXRα mRNA in m^{6}A-containing transcripts derived from HepG2 cells transfected with siFTO (n=3). (**K**) Putative m^{6}A sites in human ChREBP mRNA and the fragment used for the cloning of the reporter. (**L**) The LXRα mRNA decay in Hepa1-6 cells with Ad-FTO infection (n=3). (**M**) Relative mRNA levels and protein levels of ChREBP in Ad-FTO-infected HepG2 cells cultured under low-glucose condition (Low Glc) (n=3). (**N**) Relative mRNA levels of FTO in HepG2 cells treated with Ad-FTO and siChREBP (n=3). Means ±SEM are shown. *p<0.05, **p<0.01, ***p<0.001. NS denotes not significant.

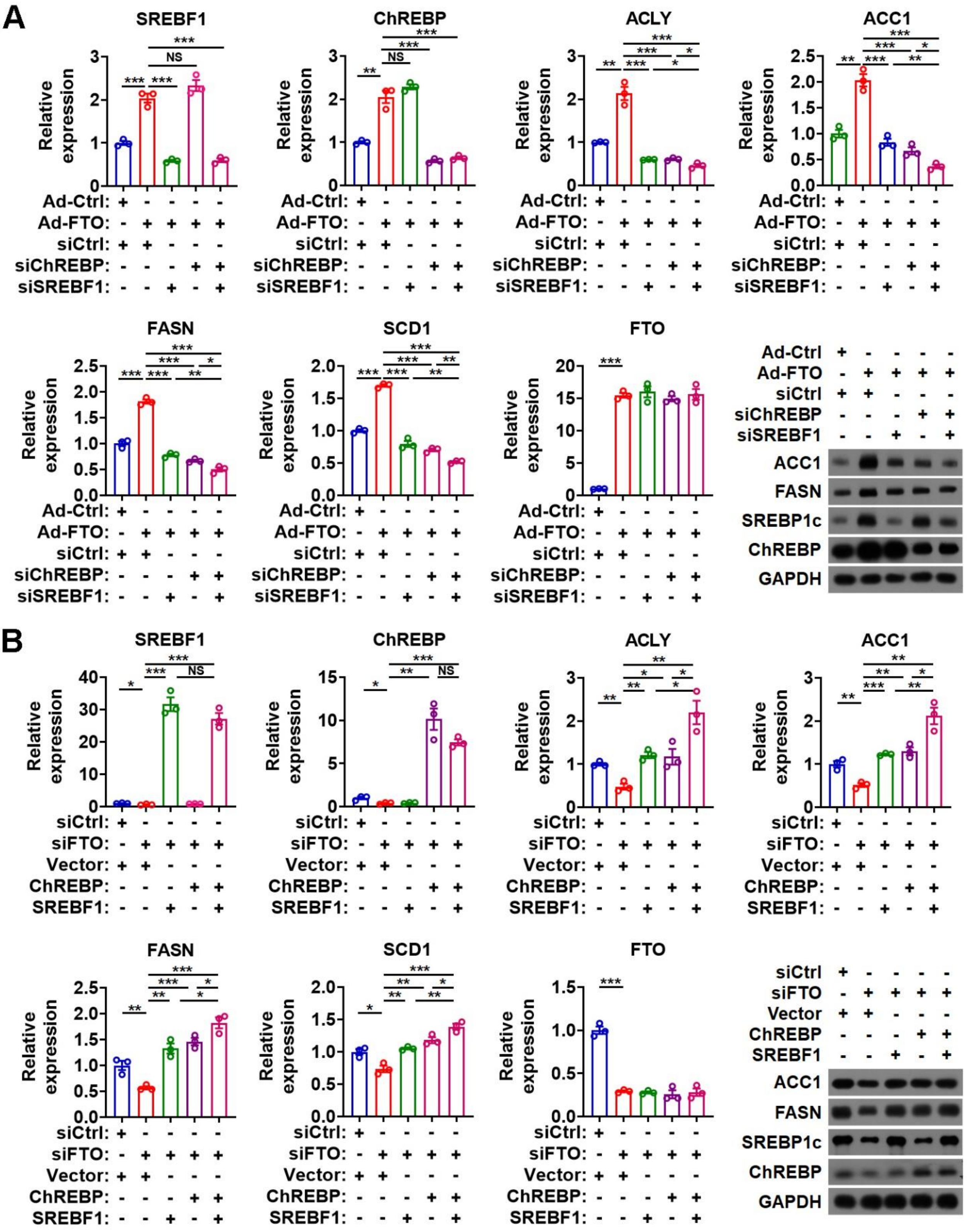

Supplementary Figure S5. Both SREBF1 and ChREBP contributes to the FTO effect on lipogenic genes. (**A**) Relative mRNA levels of SREBF1, ChREBP, FTO, and lipogenic genes and protein levels of ACC1, FASN, SREBP1c, and ChREBP in HepG2 cells treated with Ad-FTO and indicated siRNA (n=3). (**B**) Relative mRNA levels of SREBF1, ChREBP, FTO, and lipogenic genes and protein levels of ACC1, FASN, SREBP1c, and ChREBP in HepG2 cells treated with siFTO and indicated plasmid (n=3). Means±SEM are shown. *p<0.05, **p<0.01, ***p<0.001. NS denotes not significant.

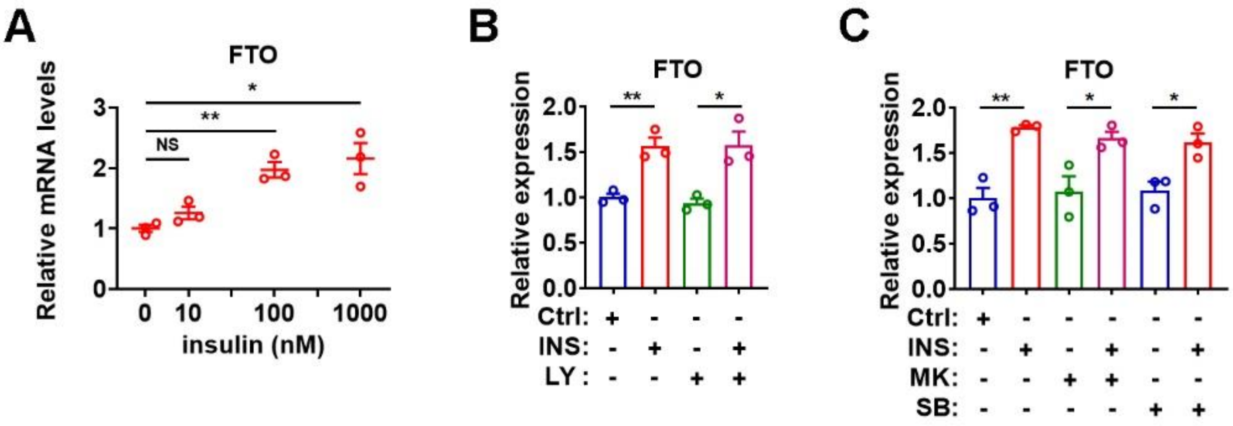

Supplementary Figure S6. The mRNA expression of FTO is regulated by insulin.

(**A**) Relative mRNA levels of FTO in HepG2 cells treated with 0, 10, 100, 1000 nM insulin (n=3). (**B**) Relative mRNA levels of FTO in HepG2 cells treated with insulin and LY294002 (n=3). (**C**) Relative mRNA levels of FTO in HepG2 cells treated with insulin, MK2206, and sb415286 (n=3). Means±SEM are shown. *p<0.05, **p<0.01, ***p<0.001. NS denotes not significant.

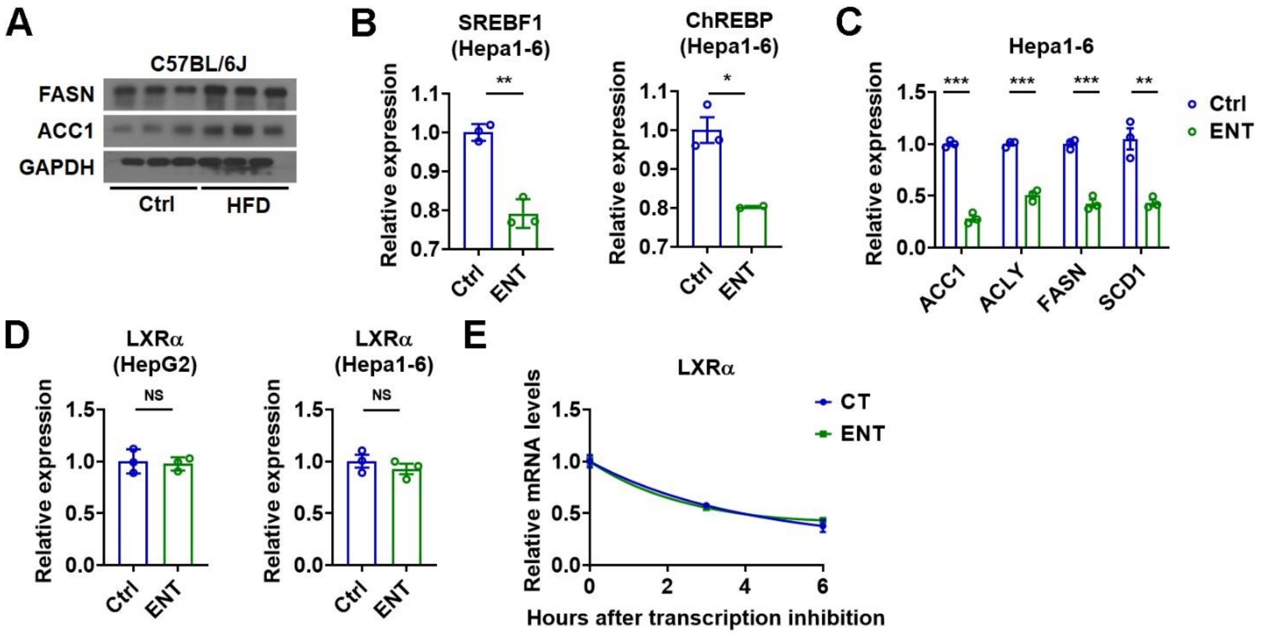

Supplementary Figure S7. FTO inhibition reduced the expression of SREBF1, ChREBP, and lipogenic genes (but not LXRα) in hepatocytes.

(**A**) Protein levels of FASN, ACC1 in the liver of HFD-fed mice (n=3). (**B**) Relative mRNA levels of SREBF1 and ChREBP in ENT-treated Hepa1-6 (n=3). (**C**) Relative mRNA levels of lipogenic genes in Hepa1-6 cells treated with ENT (n=3). (**D**) Relative mRNA levels of LXRα in ENT-treated HepG2 cells or Hepa1-6 cells (n=3). (**E**) The LXRα mRNA decay in ENT-treated Hepa1-6 cells (n=3). Means±SEM are shown. *p<0.05, **p<0.01, ***p<0.001. NS denotes not significant.

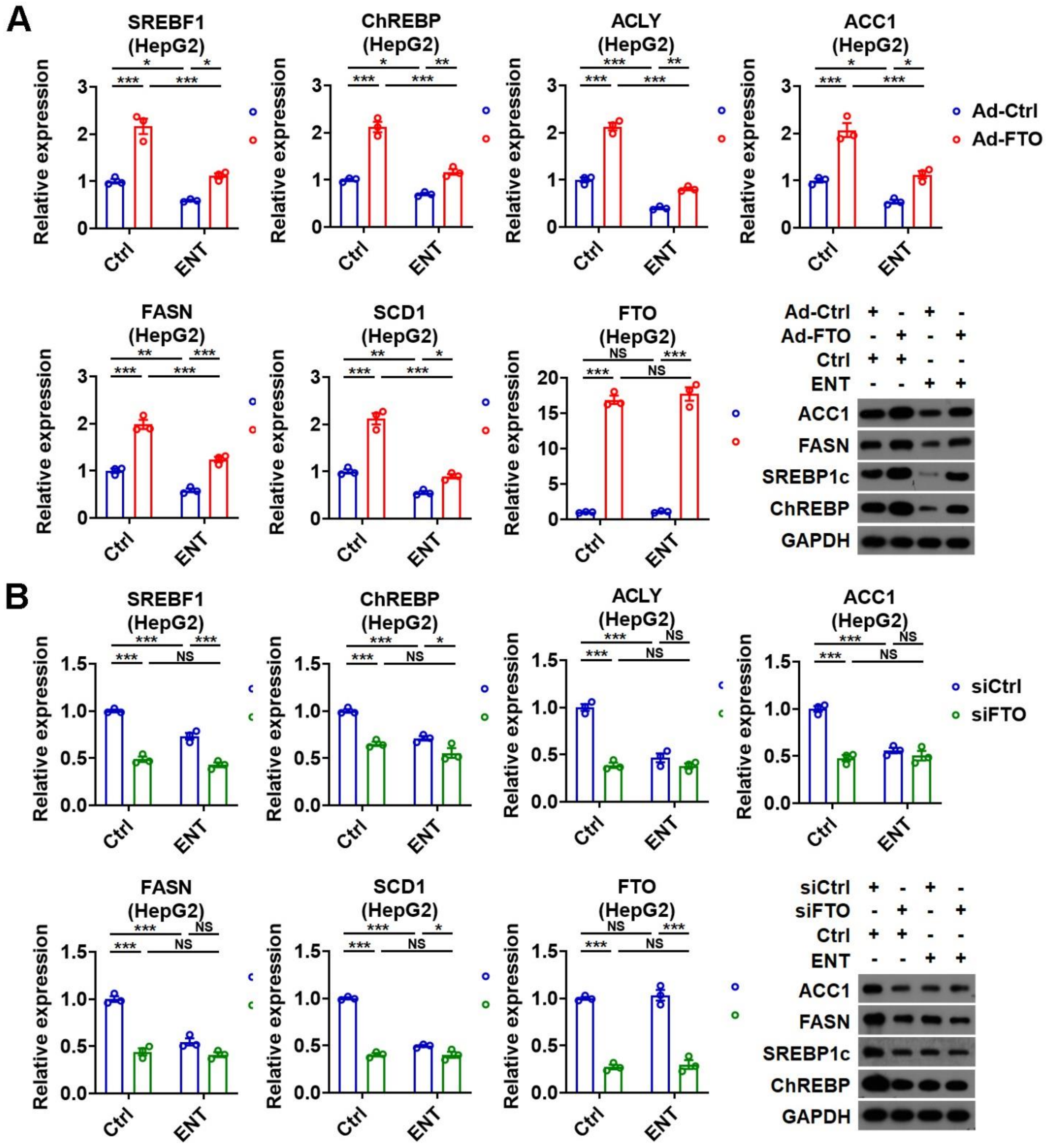

Supplementary Figure S8. The effect of ENT on lipogenic genes is attributed to FTO.

(**A**) Relative mRNA levels of SREBF1, ChREBP, FTO, and lipogenic genes, and protein levels of ACC1, FASN, ChREBP, and SREBP1c in HepG2 cells treated with Ad-FTO and ENT (n=3). (**B**) Relative mRNA levels of SREBF1, ChREBP, FTO, and lipogenic genes, and protein levels of ACC1, FASN, ChREBP, and SREBP1c in HepG2 cells treated with siFTO and ENT (n=3). Means ±SEM are shown. Statistical significance was determined by two-way ANOVA with Tukey's multiple comparisons test. *p<0.05, **p<0.01, ***p<0.001. NS denotes not significant.

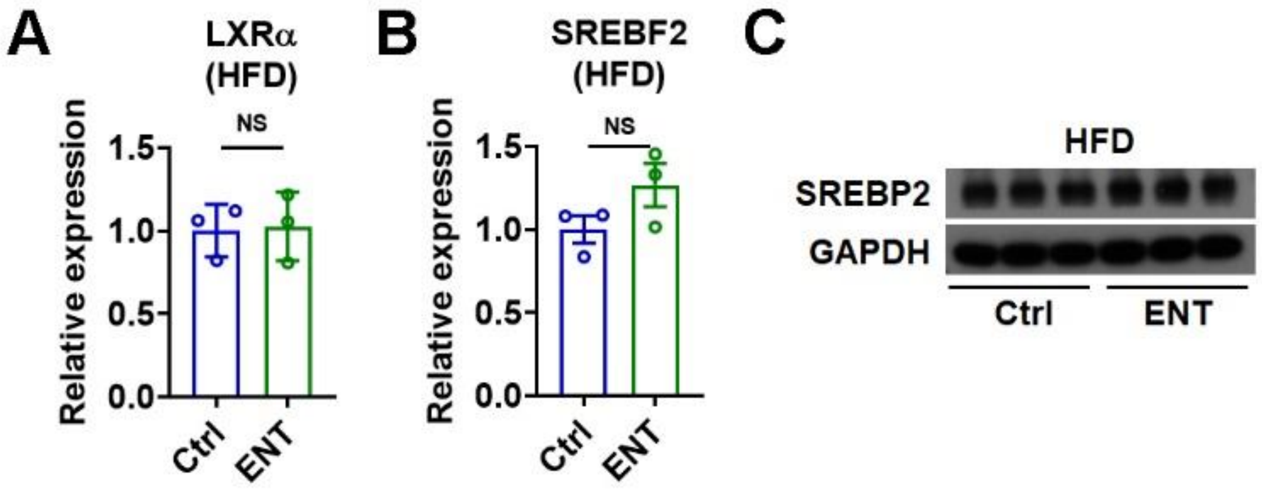

Supplementary Figure S9. ENT does not change the expression of LXRα and SREBF2 in HFD-fed mice. (**A**) Relative mRNA levels of LXRα in the liver of ENT-treated HFD-fed mice (n=3). (**B** and **C**) Relative mRNA levels of SREBF2 and protein levels of SREBP2 in the liver of ENT-treated HFD-fed mice (n=3). Means ±SEM are shown. *p<0.05, **p<0.01, ***p<0.001. NS denotes not significant.

Supplementary Tables

Supplementary Table S1: Two-way ANOVA analysis for the qPCR results in Fig. S8 with ENT treatment and Ad-FTO/siFTO as main factors.

**Supplementary Figure S8A HepG2 cells treated with Ad-FTO and ENT.**					
	**SS**	**DF**	**MS**	**F (DFn, DFd)**	**P value**
**SREBF1**					
Interaction	0.3089	1	0.3089	F (1, 8) = 13.03	P=0.007
**ChREBP**					
Interaction	0.3166	1	0.3166	F (1, 8) = 24.44	P=0.001
**ACLY**					
Interaction	0.3913	1	0.3913	F (1, 8) = 50.32	P<0.001
**ACC1**					
Interaction	0.1865	1	0.1865	F (1, 8) = 7.753	P=0.02
**FASN**					
Interaction	0.07923	1	0.07923	F (1, 8) = 6.492	P=0.03
**SCD1**					
Interaction	0.446	1	0.446	F (1, 8) = 30.83	P<0.001
**FTO**					
Interaction	0.4129	1	0.4129	F (1, 8) = 0.4428	P=0.52
**Supplementary Figure S8B HepG2 cells treated with siFTO and ENT.**					
	**SS**	**DF**	**MS**	**F (DFn, DFd)**	**P value**
**SREBF1**					
Interaction	0.03236	1	0.03236	F (1, 8) = 15.06	P=0.005
**ChREBP**					
Interaction	0.02804	1	0.02804	F (1, 8) = 9.144	P=0.02
**ACLY**					
Interaction	0.2076	1	0.2076	F (1, 8) = 54.18	P<0.001
**ACC1**					
Interaction	0.1638	1	0.1638	F (1, 8) = 39.85	P<0.001
**FASN**					
Interaction	0.1337	1	0.1337	F (1, 8) = 41.15	P<0.001
**SCD1**					
Interaction	0.1895	1	0.1895	F (1, 8) = 159.5	P<0.001
**FTO**					
Interaction	4.37E-05	1	4.37E-05	F (1, 8) = 0.009106	P=0.93

Supplementary Table S2: siRNA sequences.

**siRNA**	**Sequence 5'→3'**
siCtrl	UCUCCGAACGUGUCUCUCGUCGUTT
siFTO	AAAUAGCCGCUGCUUGUGAGATT
siCtrl	UUCUCCGAACGUGUCACGUTT
siSREBF1	GCGGCUGUUGUCUACCAUATT
siCtrl	UUCUCCGAACGUGUCACGUUU
siChREBP	GCACCCUUGGCAAACCUUUUU

Supplementary Table S3: Primer sequences for plasmid construction.

**Plasmid**	**Sequence 5'→3'**
pcDNA3.1-hFTO	F:CCCTCGAGATGAAGCGCACCCCGACTGCCGAGGAAC
	R:GCTCTAGACTAGGGTTTTGCTTCCAGAAGCTGACCTC
pRL-TL SREBF1	F:GCTCTAGAGGACCACTGTCACTTCCAGCTAGACC
	R: GCGGCCGCTGCAGCCCGTGGATTCCGACC
pRL-TL ChREBP	F:GCTCTAGATGCCAGCAGCAGCTGCCCG
	R: GCGGCCGCAAACCCACACACACACATCCACACACAC
pRL-TL LXRα	F: GCTCTAGACGGAGGTACAACCCTGGGAGTGAGAG
	R: GCGGCCGCACAGGAAGTGGTGGAGGCAGGATGG

Supplementary Table S4: Primer sequences for quantitative Real-Time PCR.

**Gene**	**Sequence 5'→3'**
mFASN-(+)	CGGTATGTCGGGGAAGTTGC
mFASN-(-)	CGGAGTGAGGCTGGGTTGATA
mACC-(+)	GAGACGCTGGTTTGTAGAAGT
mACC-(-)	TCGCTGGGTGGGTGAGATG
mB-ACTIN-(+)	CTTCTTTGCAGCTCCTTCGTT
mB-ACTIN-(-)	AGGAGTCCTTCTGACCCATTC
mSCD1-(+)	TTCTTGCGATACACTCTGGTGC
mSCD1-(-)	CGGGATTGAATGTTCTTGTCGT
mGAPDH-(+)	ACATCATCCCTGCATCCACT
mGAPDH-(-)	GTCCTCAGTGTAGCCCAAG
mACLY-(+)	ACCCTTTCACTGGGGATCACA
mACLY-(-)	GACAGGGATCAGGATTTCCTTG
mSREBF1-(+)	TGACCCGGCTATTCCGTGA
mSREBF1-(-)	CTGGGCTGAGCAATACAGTTC
mChREBP-(+)	AGATGGAGAACCGACGTATCA
mChREBP-(-)	ACTGAGCGTGCTGACAAGTC
mLXRα-(+)	CTCAATGCCTGATGTTTCTCCT
mLXRα-(-)	TCCAACCCTATCCCTAAAGCAA
mCPT1-(+)	AGATCAATCGGACCCTAGACAC
mCPT1-(-)	CAGCGAGTAGCGCATAGTCA
mPPARa-(+)	AGAGCCCCATCTGTCCTCTC
mPPARa-(-)	ACTGGTAGTCTGCAAAACCAAA
mCD36-(+)	ATGGGCTGTGATCGGAACTG
mCD36-(-)	ATGGGCTGTGATCGGAACTG
mAPOB-(+)	TTGGCAAACTGCATAGCATCC
mAPOB-(-)	TCAAATTGGGACTCTCCTTTAGC
mMTTP-(+)	CTCTTGGCAGTGCTTTTTCTCT
mMTTP-(-)	GAGCTTGTATAGCCGCTCATT
mFTO-(+)	TTCATGCTGGATGACCTCAATG
mFTO-(-)	GCCAACTGACAGCGTTCTAAG
m18S-(+)	ACCGCAGCTAGGAATAATGGA
m18S-(-)	CAAATGCTTTCGCTCTGGTC

mSREBF2-(+)	GCAGCAACGGGACCATTCT
mSREBF2-(-)	CCCCATGACTAAGTCCTTCAACT
hFASN-(+)	AAGGACCTGTCTAGGTTTGATGC
hFASN-(-)	TGGCTTCATAGGTGACTTCCA
hACC-(+)	ATGTCTGGCTTGCACCTAGTA
hACC-(-)	CCCCAAAGCGAGTAACAAATTCT
hFTO-(+)	ACTTGGCTCCCTTATCTGACC
hFTO-(-)	TGTGCAGTGTGAGAAAGGCTT
hSCD1-(+)	TCTAGCTCCTATACCACCACCA
hSCD1-(-)	TCGTCTCCAACTTATCTCCTCC
hGAPDH-(+)	ATGGGGAAGGTGAAGGTCG
hGAPDH-(-)	GGGGTCATTGATGGCAACAATA
hACLY-(+)	TCGGCCAAGGCAATTTCAGAG
hACLY-(-)	CGAGCATACTTGAACCGATTCT
hSREBF1-(+)	ACAGTGACTTCCCTGGCCTAT
hSREBF1-(-)	GCATGGACGGGTACATCTTCAA
hChREBP-(+)	ACAAAAAGCGGCTCCGTAAGTCC
hChREBP-(-)	GGGGGCGGTAATTGGTGAAGAAA
hLXRα-(+)	ACACCTACATGCGTCGCAAG
hLXRα-(-)	GACGAGCTTCTCGATCATGCC
hSREBF2-(+)	CCTGGGAGACATCGACGAGAT
hSREBF2-(-)	TGAATGACCGTTGCACTGAAG

Supplementary Table S5: Primary antibodies.

**Name**	**Manufacturer**	**Art.no**	**Dilutions**	**Host**	**RRID**
FASN	CST	3180S	1:1000	Rabbit	AB_2100796
ACC	CST	3662S	1:1000	Rabbit	AB_2219400
SREBP-1	Santa Cruz	sc-8984	1:500	Rabbit	AB_2194223
MLXIPL	Proteintech	13256-1-AP	1:2000	Rabbit	AB_2266665
FTO	CST	45980	1:1000	Rabbit	AB_2799294
P-IRβ	CST	3024S	1:1000	Rabbi	AB_331253
IRβ	CST	3018S	1:1000	Rabbit	AB_560943
YTHDF2	proteintech	24744-1-AP	1:10000	Rabbit	AB_2687435
YTHDF1	proteintech	17479-1-AP	1:2000	Rabbit	AB_2217473
METTL3	proteintech	15073-1-AP	1:1000	Rabbit	AB_2142033
METTL14	Abcom	ab98166	1:1000	Rabbit	AB_10672570
SREBF2	Proteintech	28212-1-AP	1:2000	Rabbit	AB_2881091
GAPDH	proteintech	10494-1-AP	1:10000	Rabbit	AB_2263076
